# Multifunctional Cu-doped Mn_3_O_4_ nanozyme hydrogel microspheres for oral targeted treatment of inflammatory bowel disease

**DOI:** 10.1016/j.mtbio.2026.102932

**Published:** 2026-02-13

**Authors:** Wei Fan, Yinyin Chen, Wenshuang Chen, Zisong Gao, Zhongke Yang, Hongyan Li, Aimin Wu, Xianxiang Wang

**Affiliations:** a College of Science, Sichuan Agricultural University, Chengdu, Sichuan, 611130, China; b College of Agronomy, Sichuan Agricultural University, Chengdu, Sichuan, 611130, China; c Veterinary Pharmacy in College of Animal Science and Technology of Jiangxi Agricultural University, Nanchang, JiangXi, 330045, China; d Dafeng Street Taiping Community Health Service Center, Chengdu, Sichuan, 610504, China; e Institute of Animal Nutrition, Sichuan Agricultural University, Chengdu, 611130, China

**Keywords:** Cu-doped Mn_3_O_4_ nanozyme, Hydrogel microspheres, Colon targeting, Reactive oxygen species, Ferroptosis, Gut microbiota

## Abstract

Oral drug therapy for inflammatory bowel disease (IBD) is often hindered by inadequate targeting, low bioavailability, and reactive oxygen species (ROS) accumulation. To address these challenges, we have developed hydrogel microspheres@Cu-Mn_3_O_4_ nanozymes (HMCM) by encapsulating hydrothermally synthesized Cu-doped Mn_3_O_4_ nanozymes (CM NZs) into calcium alginate hydrogel microspheres (HM) using microfluidics. These microspheres are designed for colon-targeted IBD treatment. The CM NZs exhibit exceptional superoxide dismutase (SOD), catalase (CAT), and glutathione peroxidase (GPx) activities, effectively scavenging ROS such as H_2_O_2_, ·OH, and O_2_^−·^. The negatively charged HMCM promotes targeted accumulation in inflamed colon regions and facilitates specific nanozyme release. In a dextran sulfate sodium (DSS)-induced colitis mouse model, HMCM administration enhanced the expression of tight junction proteins (Claudin, ZO-1, Occludin) and repaired the damaged intestinal barrier. The oral HMCM group significantly reduced inflammation, enhanced antioxidant activity, and inhibited ferroptosis in colonic tissue through the upregulation of GPX4 and SLC7A11. It also restored gut microbiota balance by increasing probiotic populations and suppressing harmful bacteria. Systemic biosafety assessments confirmed HMCM's colon-specific retention and high biocompatibility. This research establishes HMCM as a precision-targeted colonic drug delivery platform, offering a promising therapeutic strategy for the treatment of inflammatory bowel disease.

## Highlights

• The synthesized Cu-Mn_3_O_4_ nanozymes (CM NZs) exhibit stronger multi-enzyme mimicking activities (SOD, CAT, GPx) and free radical scavenging ability compared to Mn_3_O_4_ NPs.
• A colon-targeted composite of hydrogel microspheres@Cu-Mn3O4 nanozymes (HMCM) was engineered for precise oral nanozyme delivery.
• HMCM treats IBD via a triple-synergistic mechanism. It simultaneously reduces inflammation and oxidative stress, inhibits ferroptosis by upregulating the *GPX4*/*SLC7A11* pathway, and beneficially reshapes the gut microbiota, collectively restoring intestinal homeostasis.
• HMCM demonstrates excellent systemic biosafety and colon-specific retention.

## Graphical abstract

Calcium alginate hydrogel microspheres @ Cu-Mn_3_O_4_ nanozymes (HMCM) were successfully synthesized. Due to their negatively charged surface, HMCM achieves site-specific enrichment in the colon and releases Cu-Mn_3_O_4_ nanozymes (CM NZs), which exhibit antioxidant enzyme-like activity and reactive oxygen species (ROS) scavenging capacity. In a DSS-induced colitis mouse model, HMCM effectively treated IBD through a triple synergistic mechanism: enhancing antioxidant and anti-inflammatory activities, inhibiting ferroptosis in colonic tissue cells, and regulating gut microbiota. This work presents an efficient, targeted strategy for IBD therapy.

**Figure:**
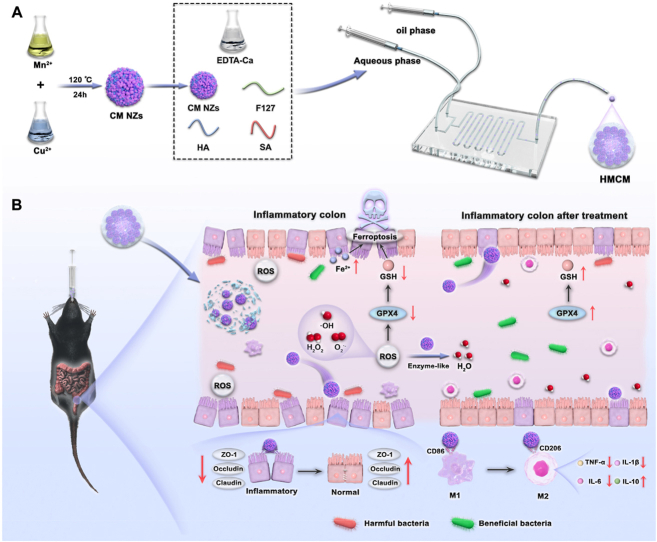

## 1 Introduction

Inflammatory bowel disease (IBD) refers to a group of chronic intestinal inflammatory disorders [[1], [2], [3]]. Typical symptoms include abdominal pain, rectal bleeding, persistent diarrhea, fatigue, and weight loss [4,5]. With over 6.8 million people affected worldwide, IBD represents a significant global health burden [6,7]. Its pathogenesis remains incompletely understood [[8], [9], [10], [11]]. Current treatments aim to relieve symptoms, control disease progression, and improve quality of life, as no curative therapy currently exists. Oral anti-inflammatory drugs are the preferred first-line option for IBD patients due to ease of self-administration and high patient compliance [12,13]. However, this route of administration faces numerous challenges: such as insufficient drug targeting and retention within the inflamed colon, limited absorption at the site of inflammation, and difficulty counteracting excessive production of pro-inflammatory cytokines within the colon—all of which limit the efficacy of oral administration [[14], [15], [16]]. To address these limitations, researchers have explored innovative nanotechnology solutions. For instance, one study demonstrated the self-assembly of nanoparticles using chitosan and glycyrrhizic acid, which have mucosal adhesion properties, thereby enhancing retention time in inflamed intestines [17]. Other studies have used the outer membrane vesicles of *Stenotrophomonas maltophilia* together with borneol to create hybrid liposomes capable of targeting and delivering luteolin to inflamed colon areas [18]. Additionally, some reports have utilized a neutrophil-macrophage hybrid membrane to coat Prussian blue nanozymes, facilitating their targeted delivery to sites of intestinal inflammation, thereby inhibiting the excessive production of pro-inflammatory factors [19].

Nanozymes show great promise for treating IBD due to their multi-enzyme mimetic activities (e.g., SOD, CAT, GPx, POD), antioxidant properties, and good biocompatibility [20,21]. They mitigate inflammation through scavenging ROS [22,23], regulating macrophage polarization [24], inhibiting NLRP3 inflammasomes [25], restoring the intestinal barrier [26] and enhanced anti-inflammatory. At the same time, nanozymes possess the advantages of material stability and functionalization (such as loading drugs or photothermal agents). Therefore, nanozymes have attracted much attention in the treatment of IBD. However, oral administration of nanozymes faces challenges such as inactivation under gastrointestinal conditions and are rapidly cleared colon mucosal flushing and peristalsis, leading to short retention and inadequate accumulation at inflamed sites [20,27]. To address these limitations, current research is focusing on several strategies. First, with respect to mucin penetration and adhesion modification, surface modification techniques can enhance the capability of nanozymes to penetrate the mucin layer, while bioadhesive materials can prolong the retention of nanozymes at inflammatory sites [[28], [29], [30]]. Second, in the realm of biomimetic delivery using biological membranes, encapsulating nanozymes in biological membranes can help them evade immune system recognition, reducing uptake by the reticuloendothelial system, extending circulation time, and facilitating targeting of inflammatory sites [31]. Third, in terms of microenvironment responsiveness, intelligent responsive materials are being designed to leverage the stomach and intestinal microenvironment, thereby overcoming barriers to adequate passage through these regions [32]. Some studies have reported using pH-sensitive calcium alginate hydrogel microspheres to assemble MnO_2_ nanozymes, berberine, and cell-penetrating peptide-modified magnolol liposomes. These components protect active ingredients from gastric acid and release them in a targeted manner in the intestine, thereby achieving a “cocktail” strategy for the treatment of ulcerative colitis [33]. Hydrogel microspheres offer significant advantages, such as small size, injectability, and controllable release, effectively overcoming the limitations of traditional delivery methods, including degradation in stomach acid and rapid clearance in the intestine. Their porous structure protects nanozymes from degradation by digestive enzymes and enables sustained, targeted release at inflammatory sites [34]. Recent studies highlight their suitability as nanozyme delivery vehicles for disease treatment [35]. Consequently, integrating nanozymes into hydrogel microspheres to create “nanozyme-hydrogel microsphere” composites is considered an ideal solution for addressing the delivery challenges associated with conventional oral nanozyme formulations.

In this study, microfluidic technology was utilized to combine copper-doped Mn_3_O_4_ nanozymes (CM NZs) with calcium alginate hydrogel microspheres (HM), resulting in a colon-targeted copper-manganese nanozyme smart hydrogel microsphere, denoted as hydrogel microspheres@Cu-Mn_3_O_4_ NZs (HMCM). The CM NZs component in this complex demonstrates remarkable superoxide dismutase (SOD), catalase (CAT), and glutathione peroxidase (GPx) activities, effectively scavenging reactive oxygen species (H_2_O_2_, ·OH, and O_2_^−·^). Additionally, the negatively charged surface of HMCM facilitates targeted delivery to inflamed regions of the colon. Importantly, HMCM ensures the specific release of CM NZs within the colonic fluid.

**Scheme 1:**
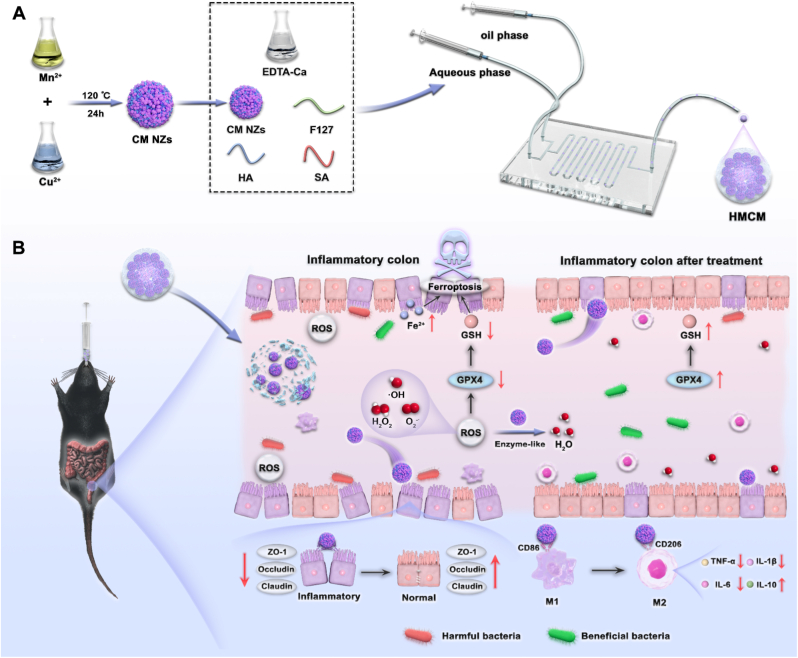
Schematic illustration of HMCM preparation and mechanism for IBD treatment.(A) Schematic diagram detailing the synthesis of CM NZs and the preparation of hydrogel microsphere loading and (B) Schematic illustration of HMCM-mediated targeted therapy for IBD, employing triple-action synergistic mechanisms: ROS scavenging, ferroptosis inhibition, and remodeling of the gut microbiota.

This system is capable of treating inflammatory bowel disease (IBD) through a triple synergistic mechanism: enhancing antioxidant and anti-inflammatory activities, inhibiting ferroptosis in colonic tissue cells, and reshaping the gut microbiota, as illustrated in Scheme 1.

## 2 Materials and methods

### 2.1 Synthesis of Cu-doped Mn_3_O_4_ nanozymes (CM NZs)

Based on existing laboratory research [36], CM NZs were synthesized via hydrothermal doping: Mn(Ac)_2_ (3.0 mmol) and varying amounts of Cu(Ac)_2_ (1.2, 1.5, 2.0, or 3.0 mmol) were dissolved in 60 mL anhydrous ethanol under magnetic stirring. The mixture was reacted at 120 °C for 24 h in a 100 mL reactor. After cooling, products were washed sequentially with ethanol/water and dried vacuum.

### 2.2 Preparation of hydrogel microspheres (HM) and loading of CM NZs (HMCM)

The preparation of HM employs a microfluidic method. First, 2 wt% hyaluronic acid, 10 wt% Pluronic F-127, and 2 wt% sodium alginates are stirred and dissolved in water. Next, a 3 M CaCl_2_ solution is mixed with a 0.5 M EDTA solution to prepare a neutral Ca-EDTA solution (solution B) with a concentration of approximately 120 mM. Solution A and solution B are then mixed in equal proportions to form the aqueous phase. The acetic acid-containing droplet-generation oil was used as oil phase. Subsequently, at 4 °C, oil phase (50 μL/min) and aqueous phase (20 μL/min) co-injected to form dispersed phase. After stopping the collection, the mixture is allowed to solidify for approximately 15 min, then washed with PBS to obtain HM. To prepare for HMCM, simply add 1 mg/mL of CM NZs to Solution A, with the subsequent steps identical to those for HM preparation.

### 2.3 Determination of the loading rate of CM NZs in HM

After microwave digestion of CM NZs and HMCM, ICP-MS was used to determine the Cu and Mn content in CM NZs and HMCM.

### 2.4 Enzyme-like activity of CM NZs

Enzyme activity of CM NZs at different concentrations was tested using SOD, CAT, and GPx enzyme activity assay kits according to the corresponding instructions.

### 2.5 ·OH radical scavenging activity of CM NZs

CM NZs' ·OH scavenging capacity was quantified via methylene blue (MB) capture [37]: Reaction mixtures containing 0.2 M NaAc-HAc buffer (2.3 mL), MB (50 μg/mL, 200 μL), H_2_O_2_ (20 mM, 200 μL), FeSO_4_ (10 mM, 200 μL), and CM NZs (100 μg/mL, 100 μL) were incubated at RT. UV-vis spectra were recorded immediately post-reaction. Parallel ·OH detection used EPR with DMPO trapping.

### 2.6 O_2_^−·^ scavenging activity of CM NZs

O_2_^−·^ scavenging activity was used by NBT reduction [38]: In PBS buffer (2.0 mL), add L-Met (13 mM, 200 μL), EDTA-Na_2_ (1 mM, 300 μL), VB_2_ (20 μM, 200 μL), NBT (37.5 μM, 300 μL), and CM NZs (0-50 μg/mL, 200 μL). After 10-min light exposure, centrifuge at 21,000 rpm (10 min) and measure the absorbance at 560 nm. Parallel O_2_^−·^ detection used EPR with DMPO trapping. Activity was calculated as:

(1) $${O_{2}}^{\cdot -}\text{scavenging}\;\text{rate}=\frac{\left(A_{0}-A_{n}\;\right)}{A_{0}}\times 100\%$$

where A_0_ represents the control absorbance and A_n_ represents the sample absorbance.

### 2.7 H_2_O_2_ scavenging activity of CM NZs

The scavenging activity of CM NZs toward H_2_O_2_ was determined using the terephthalic acid (TA) method [39]. The specific procedure is as follows: Take 150 μL of H_2_O_2_ (10 mM) and 150 μL of CM NZs (25-150 μg/mL), add 2.6 mL of PBS buffer, and mix in the dark for 1 h. The resulting solution is mixed with 50 μL FeSO_4_ (1 mM) for 30 min, then 50 μL TA solution (30 mM) is added. After 60 min in the dark, fluorescence emission is measured using a fluorescence spectrometer (Ex = 320 nm, Em = 425 nm). Activity was calculated as:

(2) $$H_{2}O_{2}\;\text{scavenging}\;\text{rate}=\frac{F_{0}-F_{n}}{F_{0}}\times 100\%$$

where F_0_ is the control fluorescence intensity, and F_n_ is the sample fluorescence intensity.

### 2.8 DPPH radical scavenging activity of CM NZs

DPPH radical scavenging capacity was assessed by measuring absorbance decay at 517 nm [40]. Samples containing 0.1 mM DPPH and CM NZs (varying concentrations) were incubated in dark (RT, 15 min). After instantaneous centrifugation, the supernatant was taken to determine the absorbance was measured spectrophotometrically. Activity was calculated as:

(3) $$\text{DPPH}\;\text{scavenging}\;\text{rate}=\frac{A_{0}-A_{n}}{A_{0}}\times 100\%$$

### 2.9 ABTS ^+^ radical scavenging activity of CM NZs

The determination of ABTS^+^ radical scavenging activity was performed using a previously reported method [41]. Specifically, 7.4 mM ABTS reserve solution with 2.6 mM K_2_S_2_O_8_ in the dark for 16 h to generate ABTS ^+^ radicals. Subsequently, the ABTS^+^ solution was diluted with PBS to an absorbance value of 0.7-0.8 at 734 nm and set aside. In the experiment, 200 μL of CM NZs solution (25-150 μg/mL) was mixed with 1.8 mL of ABTS^+^ solution and incubated in the dark for 3 min. After instantaneous centrifugation, the supernatant was taken to determine the absorbance at 734 nm was then measured using a UV-visible spectrophotometer. Activity was calculated as:

(4) $${\text{ABTS}}^{+}\;\text{scavenging}\;\text{rate}=\frac{A_{0}-A_{n}}{A_{0}}\times 100\%$$

### 2.10 Cell viability

Cytotoxicity of CM NZs, HM, and HMCM was evaluated in Hepatocytes(Hep), Mouse colon epithelial cells (MCEC), and Mouse Mononuclear Macrophages Cells cells (RAW264.7) via CCK-8 assay: Seed 5 × 10^4^ cells in 96-well plates 24 h,add CM NZs in fresh medium, incubate 12 h add 10 μL CCK-8 incubate 1 h, the absorbance at 450 nm was measured using a microplate reader.

### 2.11 HMCM resistant to gastric juice and targeted colon release CM NZs

*In vitro* investigation: The amount of CM NZs in HMCM was determined using ICP-MS. Subsequently, CM NZs (1 mg/mL) and HMCM containing an equal amount of CM NZs were sequentially added in simulated gastric fluid (SGF), simulated intestinal fluid (SIF), and simulated colonic fluid (SCF). At different times, the supernatant from each simulated fluid was collected and analyzed using ICP-MS to determine the concentrations of Cu and Mn. Additionally, the HMCM in the simulated fluids was washed, centrifug and collected. The collected samples were examined using SEM to observe changes in their appearance.

*In vivo* tracking: Male C57BL/6 mice (8-week-old) were administered 3% DSS to establish colitis models. Colitis-induced (model group) and healthy (control group) mice received oral gavage of Cy5-labeled HMCM. After 12 h, gastrointestinal distribution was analyzed using an *in vivo* imaging system (AniView Kirin SE, China), with fluorescence signals quantified along the digestive tract.

### 2.12 Intracellular ROS and lipid ROS scavenging capacity

RAW 264.7 cells were primed with LPS for 12 h, followed by 12 h treatment with CM NZs, HM, or HMCM. Cells were then stained with 20 μM H_2_DCFDA (total ROS probe) and 5 μM C11-BODIPY (lipid ROS sensor) for 30 min. After PBS washing, perform quantitative testing by flow cytometry (BD FACS Verse, USA). Data analysis utilized FlowJo V10.

### 2.13 Macrophage M1/M2 polarization

To assess macrophage polarization, RAW 264.7 cells (2 × 10^6^ cells/well in 6-well plates) were: First, treated with 500 ng/mL LPS induce cell production M1 induction, followed by Incubated 12 h with CM NZs, HM, and HMCM, then blocked and stained with anti-CD86 (*M1* marker) and anti-CD206 (*M2* marker) antibodies, finally analyzed by flow cytometry.

### 2.14 Analysis of intracellular ROS, lipid ROS and Fe^2+^ in MECE cells

1.0 × 10^5^ MCEC cells were seeded into a 12-well plate and cultured for 24 h to promote cell adhesion. First, ferroptosis was induced MCEC cells with 1.5 μM RSL3. Then, cells from different groups were all incubated for 12 h. The cells were further labeled with 20 μM H_2_DCFDA, 5 μM CD11-BODIPY, and 1 μM Far-Red Labile Fe^2+^ probe for 30 min, PBS was washed by flow cytometry.

### 2.15 Measurement of glutathione (GSH) and malondialdehyde (MDA) content

1 × 10^5^ MCEC cells were cultured in 6-well plates and treated according to the *2.14* method. Cellular total glutathione (T-GSH) and malondialdehyde (MDA) levels were quantified using commercial assay kits per manufacturer's protocols.

### 2.16 *In vivo* biocompatibility evaluation of HMCM

To evaluate HMCM biocompatibility, 8-week-old C57BL/6 mice received oral gavage of HMCM (10 mg/kg) and equal-volume saline. Blood was collected at days 1, 7, and 14 post administration for blood test and serum biochemistry (AST/ALT). Major organs (heart, liver, spleen, lung, kidney) were collected for H&E staining and Elemental quantification (Cu and Mn). The 14-day HMCM group was designated for chronic toxicity assessment.

### 2.17 Therapeutic effect of HMCM in a DSS-induced colitis model

Six-week-old male C57BL/6 mice (18-20g) were purchased from Chengdu Dashuo Experimental Animal Co., LTD. Throughout the experiment, mice were housed under controlled conditions ranging from 23 to 27 °C, 45% to 65% relative humidity, and a 12-h light/dark cycle. All aspects of routine animal care and experimental procedures were performed in accordance with guidelines approved by the Animal Ethics Committee of Sichuan Agricultural University. (License No. SICAU 2024-06-159). After one week of acclimatization, the mice were divided into five groups: i) Control (200 μL of normal saline),ii) DSS (150 μL of normal saline),iii) DSS + CM NZs (10 mg/kg), iv) DSS + HM (36 mg/kg), v) DSS + HMCM (36 mg/kg). Group ii-v received 3%DSS solution through drinking water for 7 days to induce inflammatory enteritis, and then changed to drinking water. On the 8th day, the treatment was continuously given for 3 times according to the group scheme. Throughout the treatment period, A colitis disease activity index (DAI) changes in weight loss, stool viscostiy, hematochezia, physicial condition, and activity level were recorded (See Table S1 for details) [42]. The DAI scoring was performed by investigators who were not involved in the animal grouping and treatment administration. After the treatment, the mice were anesthetized, blood was collected for a complete blood count and serum separation. Colon tissue was extracted and examined via H&E staining, immunohistochemistry (IHC), immunofluorescence staining, and tissue TEM. Additionally, the expression levels of cytokines (TNF-α, IL-1β, IL-6, IL-10, GPX4, CD98, and SLC7A11) in colon tissue were detected by qRT-PCR (primer sequences are detailed in Table S2). IHC was used to detect the expression of tight junction proteins (ZO-1, Occludin, and Claudin) and ferroptosis marker proteins (GPX4). Immunofluorescence staining was used to label CD86/CD206 to distinguish *M1*/*M2*-type macrophages. TEM sections of tissue were used to observe the morphology of epithelial villi and mitochondria in colonic tissue cells.

### 2.18 Intestinal microbiota analysis

Fecal samples were aseptically collected from three groups. Total genomic DNA was extracted using ZymoBIOMICS DNA Microprep Kit (D4301). The 16S rRNA genes (V4 region) was amplified with primers 515F (5′-GTGYCAGCMGCCGCGGTAA-3′) and 806R (5′-GGACTACHVGGGTWTCTAAT-3′). Amplicon sequencing was performed on Illumina MiSeq platform with standard bioinformatic pipelines.

### 2.19 Statistical analysis

Data are presented as mean ± SD. Intergroup differences were analyzed using Student's t-test (two-group comparisons) or one-way ANOVA (multi-group comparisons). Statistical significance was indicated as ∗*p < 0.05*, ∗∗*p < 0.01*, ∗∗∗*p < 0.001*, and ∗∗∗∗*p < 0.0001*).

## 3 Results and discussion

### 3.1 Synthesis and characterization of CM NZs

**Fig. 1:**
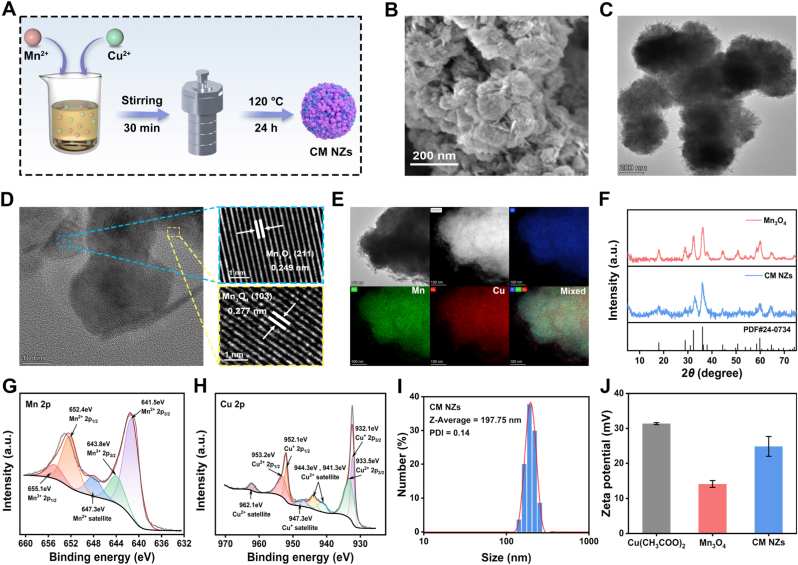
Synthesis and characterization of CM NZs. (A) Schematic of CM NZs synthesis. (B) SEM, (C) HRTEM, (D) Lattice constant and (E) Elemental mapping of CM NZs. (F) XRD of Mn_3_O_4_ and CM NZs (PDF#24-0734). XPS spectra of the (G) Mn 2p and (H) Cu 2p region. (I) DLS and PDI values of CM NZs. (J) Zeta Potential of Cu (CH_3_COO)_2_, Mn_3_O_4_ and CM NZs.

Building upon previous Mn_3_O_4_ synthesis studies [36], CM NZs were synthesized using a simplified one-pot hydrothermal method, as illustrated in Fig. 1A. Optimizing copper doping within a range of 28.5% to 50% by weight resulted in CM NZs exhibiting enhanced multi-enzyme activities, including those of CAT, SOD, and GPx. Scanning electron microscopy (SEM) indicated that higher copper content induced a morphological evolution from spherical Mn_3_O_4_ to urchin-like structures (Fig. S1A). Furthermore, X-ray diffraction (XRD) confirmed progressive crystal modifications (Fig. S1B). Enzyme activity assays identified that the variant with 40 wt% copper doping displayed optimal enzyme activity (Fig. S2). As a result, CM NZs with 40 wt% copper doping were selected for further experiments.

These nanoparticles exhibited petal-like morphology (Fig. 1B), with HRTEM revealing Mn_3_O_4_ characteristic lattice fringes at 0.249 nm (211) and 0.277 nm (103) (Fig. 1C and D), indicating Mn_3_O_4_ preserved crystal structure [43,44]. Elemental mapping confirmed homogeneous copper distribution (Fig. 1E), and ICP-MS quantified Cu/Mn content at 31.50/48.32 wt% (Fig. S3). XRD patterns matched standard Mn_3_O_4_ (PDF#24-0734) [45], further confirming that CM NZs primarily adopt a Mn_3_O_4_ crystal structure (Fig. 1F). XPS analysis (Fig. S4A) detected Mn, Cu, and O species. The O 1s XPS spectrum (Fig. S4B) exhibited a peak at 529.7 eV assignable to Mn/Cu-O bond [46,47], which the characteristic vibration peak at 500-700 cm^−1^ in the FTIR spectrum (Fig. S5) also confirms the presence of the Mn/Cu-O bond. The Mn 2p spectrum (Fig. 1G) exhibited characteristic doublets at 641.5/652.4 eV and 643.8/655.1 eV, corresponding to Mn^2+^ and Mn^3+^ species, respectively [46]. Similarly, the Cu 2p spectrum (Fig. 1H) displayed four peaks assignable to Cu^+^ (932.1/952.1 eV) and Cu^2+^ (933.5/953.2 eV) [47]. These results have confirmed the coexistence of mixed-valence states for both Mn and Cu in CM NZs. DLS analysis showed that the average particle size of CM NZs was 197.75 nm, with a PDI value of 0.19, which proved that CM NZs had a relatively uniform size distribution (Fig. 1I). Zeta potential measurements positioned CM NZs (+24.83 mV) between Cu^2+^ (+31.37 mV) and Mn_3_O_4_ (+14.11 mV) (Fig. 1J), aqueous stability and further confirming CM NZs successful synthesis.

### 3.2 The multienzyme-like antioxidant activity and ROS scavenging activity of CM NZs

**Fig. 2:**
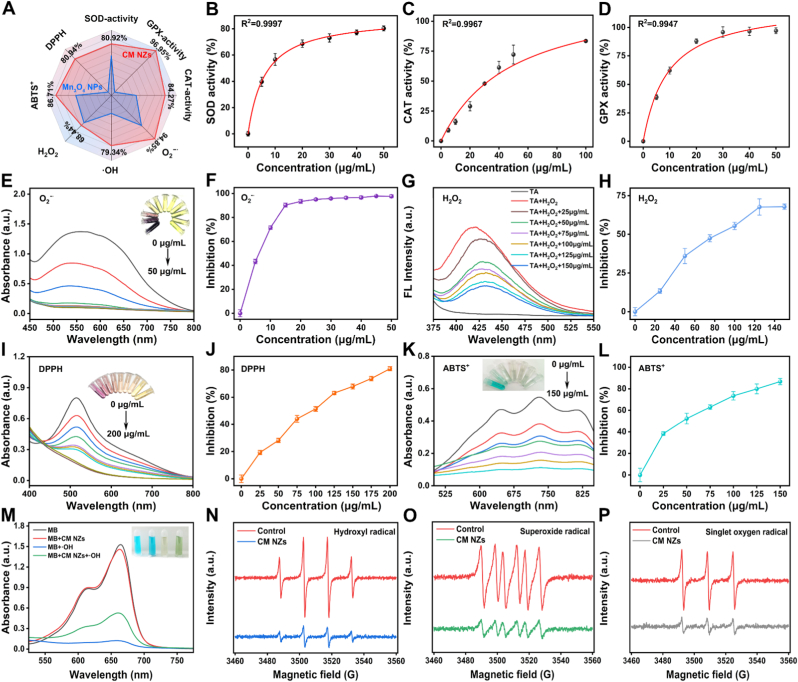
ROS scavenging and multienzyme-like antioxidative activity of CM NZs. (A) Radar map of Mn_3_O_4_ and CM NZs ROS-elimination properties, (B) SOD, (C) CAT and (D) GPX activity of CM NZs (n = 3). (E-F) O_2_^−·^, (G-H) H_2_O_2_, (I-J) DPPH, (K-L) ABTS, and (M) ·OH scavenging ability of CM NZs. (n = 3). The scavenging ability of CM NZ against (N) ·OH, (O) O_2_^·−^, and (P) ^1^O_2_ was determined by EPR.

After characterizing the nanostructure of CM NZs, we systematically evaluated their biocatalytic activity and capacity to scavenge ROS. The results indicated that CM NZs exhibited superior ROS scavenging capabilities compared to conventional Mn_3_O_4_ nanoparticles (Fig. 2A). This enhanced catalytic performance may be attributed to the Cu^+^/Cu^2+^ redox coupling system introduced through copper doping, which accelerates the Mn^2+^/Mn^3+^ electron transfer pathway and significantly enhances the catalytic rate [[48], [49], [50]]. Considering that SOD, CAT, and GPx are the primary antioxidant enzymes in living organisms, we evaluated the enzyme-like activities of CM NZs using respective assay kits. As illustrated in Fig. 2B, CM NZs demonstrated SOD-like activity at 80.92% when tested at a concentration of 50 μg/mL. CAT-like activity reached 84.27% at 100 μg/mL (Fig. 2C), while the GPx-like activity was 96.95% at 50 μg/mL (Fig. 2D). ROS, including H_2_O_2_, OH, and O_2_^−·^, are highly reactive molecules that attack vital cellular components like DNA, proteins, and lipids. Such attacks cause oxidative stress and cellular damage, disrupting normal metabolism, accelerating inflammation, and contributing to the onset and progression of various chronic diseases [51]. Our experiments have demonstrated that CM NZs possess significant ROS scavenging capacity. In the NBT assay for O_2_^−·^ scavenging [38], CM NZs (20 μg/mL) achieved a scavenging rate of 94.06% (Fig. 2E and F). The TA assay [39] for H_2_O_2_ displayed that CM NZs (120 μg/mL) effectively scavenged 68.07% of H_2_O_2_ (Fig. 2G and H). The MB assay [37] confirmed ·OH scavenging by CM NZs (Fig. 2M). Additionally, CM NZs also scavenged DPPH and ABTS^+^ radicals: at 200 μg/mL, they scavenged 80.94% of DPPH radicals (Fig. 2I and J), and at 150 μg/mL, effectively scavenged 86.71% of ABTS^+^ radicals (Fig. 2K and L). To further validate the ROS scavenging ability of CM NZs, electron spin resonance (EPR) spectroscopy using 5,5-dimethyl-1-pyrrolidine-N-oxide (DMPO) as a spin trap was employed [[52], [53], [54]]. As expected, CM NZs addition led to a significant reduction in the DMPO/·OH (Fig. 2N), DMPO/O_2_^−·^ (Fig. 2O), and DMPO/^1^O_2_ (Fig. 2P) signals. In summary, CM NZs exhibit broad-spectrum ROS scavenging capabilities, showing promise for treating oxidative stress-related diseases.

### 3.3 Preparation, characterization, and colitis-targeting effects of hydrogel microspheres loaded with CM NZs (HMCM)

**Fig. 3:**
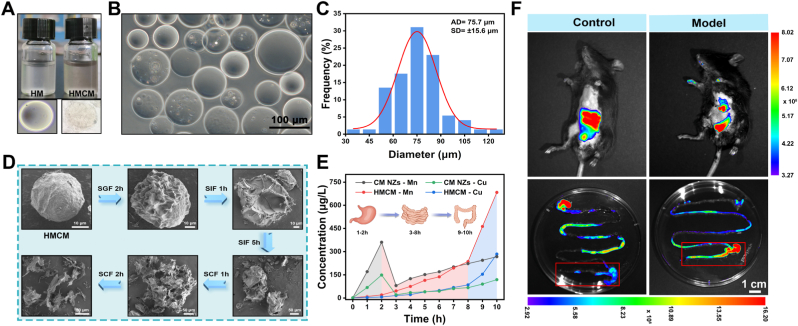
Synthesis of hydrogel microspheres and experiments on targeted adhesion to sites of intestinal inflammation. (A) HM and HMCM images at 50 mg/mL, (B) Microscopic image of HM, (C) Particle size distribution of HM, (D) SEM images after HMCM passed simulated gastric fluid (SGF), simulated intestinal fluid (SIF), and simulated colonic fluid (SCF), (E) ICP-MS determination of Cu and Mn element content in CM NZs and HMCM after passing through different simulated liquids, (F) Fluorescent imaging photographs of the entire digestive tract 12 h after oral administration of HMCM to normal mice and mice with an enteritis model.

Fig. 3A shows the fabrication of HM and HMCM using microfluidic technology in this study. To enhance the nanozyme loading efficiency in HM, CM NZs were initially coated with low-molecular-weight hyaluronic acid and Pluronic F-127. This coating occurred before synthesizing calcium alginate hydrogel microspheres on the microfluidic chip. Microscopy images (Fig. 3B and Fig. S6B) revealed that the microspheres in aqueous solution exhibited regular spherical morphology with good monodispersity and an average size of ∼75.7 μm (Fig. 3C). SEM observations further indicated their spherical structure with a reduced size of ∼30.1 μm after drying (Fig. S6A). FTIR spectral analysis indicates no significant peak position shift between the final HMCM composite and the HM matrix, confirming that CM NZs are primarily encapsulated through physical entrapment rather than the formation of new chemical bonds (Fig. S6C). Successful CM NZs loading evidenced by a 5.17 mV increased in HMCM's zeta potential versus HM (Fig. S6D). Thermal gravimetric analysis results (Fig. S6E) expressed that both HM and HMCM exhibited good thermal stability, with the difference in weight loss primarily attributed to the presence or absence of CM NZs loading. Finally, we determined the element content in HMCM by ICP-MS, among which the content of Cu element was 11.13% and the content of Mn was 16.50%. Combined with the ICP-MS measurement results of CM NZs (Cu/Mn = 0.652), the loading rate of CM NZs in HMCM was 27.63% (Fig. S6F).

Oral drug delivery for IBD faces challenges including gastric degradation and poor targeted delivery [55]. Studies have shown the abundance of cationic proteins in inflamed intestinal epithelium, anionic drug carriers offer an effective targeting strategy [56,57]. The negatively charged HMCM developed herein demonstrates potential for inflammatory colon site-specific target. To validate this, we first investigated whether the nanostructure and function of CM NZs could be impaired in the GI environment. As shown as Fig. S7A and S7B, CM NZs was uniformly dispersed in SCF and water, but there was obvious particle aggregation and sedimentation in SGF and SIF. In addition, the enzyme activity and free radical scavenging ability of CM NZs treated with SGF, SIF and SCF retained 31.4%, 82.3% and 97.8%, respectively (Fig. S7C). Subsequently, we further evaluated the performance of HM loaded with Cy5 fluorescent dye (Fig. S7D) in simulated gastrointestinal fluids. The results depicted in Fig. S7E indicate that HM remains stable in SGF, gradually swells in SIF, and ultimately ruptures to release its contents in SCF. This preliminary evidence confirms HM's resistance to gastric acid and its release characteristics within the colonic microenvironment. Further analysis, comparing CM NZs with HMCM, revealed that bare CM NZs released over 80% of copper/manganese ions in SGF, whereas HMCM maintained its structural integrity in both SGF and SIF, disintegrating only in SCF with controlled ion release (Fig. 3D and E). This finding further supports HMCM's gastric protection and colon-specific delivery functions. To assess HMCM's *in vivo* targeting ability for inflamed colons, HMCM was labeled with Cy5 and administered orally to both control mice and colitis model mice. Live imaging 12 h after administration showed a 3.2-fold increase in colonic fluorescence intensity in the colitis models compared to healthy controls (Fig. 3F), confirming HMCM's targeting capability for inflamed colon regions. In summary, HMCM enables precise delivery to inflamed colon regions with spatiotemporally controlled release, demonstrating significant potential for targeted colitis therapy.

### 3.4 Intracellular ROS and lipid ROS scavenging, and anti-ferroptosis properties

**Fig. 4:**
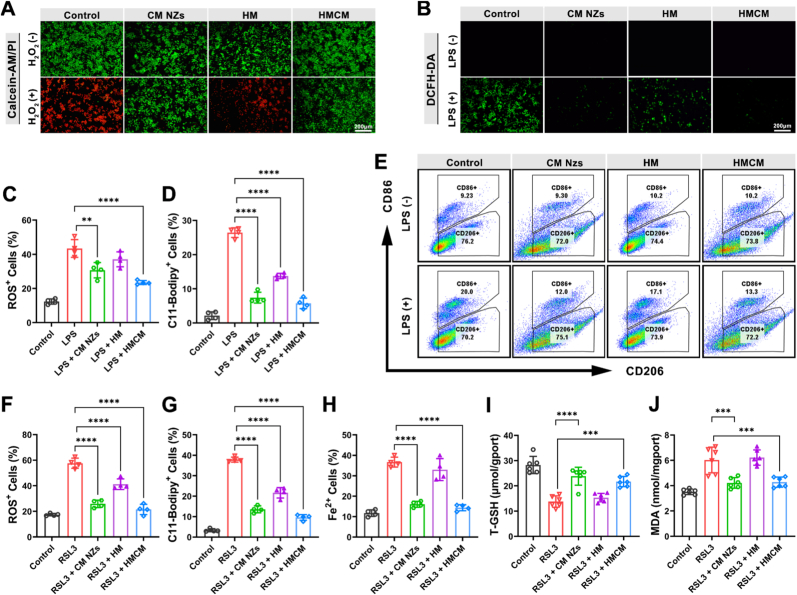
Cell experiments investigate the antioxidant and anti-ferroptosis properties of HMCM. (A) RAW 264.7 cells co-stained with Calcein-AM (live cells/green) and PI (dead cells/red). (B) CLSM image of ROS in RAW 264.7 cells. FACS for the levels of (C) ROS^+^ and (D) lipid ROS in LPS-treated RAW 264.7 cells (n = 4). (E) FACS for the polarization of RAW 264.7 cells treated under different group. FACS for the levels of (F) ROS^+^, (G) lipid ROS and (H) Fe^2+^ in RSL3-treated MCEC cells (n = 4). Levels of (I) T-GSH and (J) MDA in MCEC cells treated with RSL3 (n = 6).

To assess HMCM's scavenging capacity against intracellular ROS, we established the H_2_O_2_ model for acute oxidative stress and cell viability, and the LPS model for complex inflammatory signaling and intracellular ROS level in RAW 264.7 cells. In the H_2_O_2_ induced acute oxidative stress model (Fig. 4A), all non H_2_O_2_ treated groups maintained high viability. More than 95% of control and HM treated cells died when exposed to H_2_O_2_ alone, whereas the CM NZs group and the HMCM group retained greater over 90% cell viability post H_2_O_2_ exposure, suggesting the presence of CM NZs and HMCM could prominently protect the cells from H_2_O_2_ microenvironment *in vitro*. In the LPS-induced complex inflammatory model, ROS levels in the HM group were reduced to a certain extent, and ROS levels in CM NZs and HMCM groups were significantly lower than those in the control group, of which the HMCM group was the most significant (Fig. 4B). This superior ROS scavenging ability was attributed to the synergistic effect of the physical microenvironment provided by HMCM and the internal active components (HA and CM NZs). Subsequent flow cytometry confirmed significant reduction in ROS/lipid ROS levels in the CM NZs and HMCM groups (Fig. 4C and D and Fig. S8). Notably, the HM group exhibited significantly reduced lipid ROS levels compared to the LPS group. This effect is likely attributable to cell-microsphere interactions, where HM provides a favorable microenvironment for initial cell adhesion, growth, and spreading, thereby attenuating oxidative stress signaling pathways and ultimately suppressing lipid ROS production [58]. As is well known, controlling macrophage polarization can improve inflammation [59], we quantified *M1* (CD86^+^) and *M2* (CD206^+^) macrophage subsets across treatment groups using flow cytometry. In non-LPS-treated groups, the CD86^+^/CD206^+^ ratio remained stable, while in the LPS-treated groups, the ratio of CD86^+^/CD206^+^ in the CM NZs group and HMCM group was significantly lower than that in the control group (Fig. 4E), indicating that the CM NZs and HMCM groups can drive macrophage polarization toward a direction favorable for inflammation improvement. In summary, HMCM exhibits exceptional biocompatibility, potent ROS/lipid ROS scavenging, and the ability to effectively regulate macrophage polarization, making it is expected to be used to improve inflammatory colon disease.

During inflammation, inflammatory mediators and cytokines exacerbate oxidative stress and disrupt iron metabolism, driving lipid ROS accumulation and subsequent ferroptosis [60]. Concomitant GPX4 downregulation heightens cellular susceptibility to lipid peroxidation [61]. To investigate this axis, we established an inflammatory ferroptosis model in MCEC cells using the GPX4 inhibitor RSL3 and assessed five key indicators: ROS, lipid ROS, Fe^2+^, T-GSH, and MDA. The results showed that compared to only RSL3 treatment, CM NZs and HMCM groups demonstrated significant reduction in ROS, lipid ROS, and Fe^2+^ (Fig. 4F–H and Fig. S9A–S9C). In the ferroptosis model of MCEC cells induced by rsl3, the CM NZs and HMCM treatment groups exhibited comparable cell viability to the ferroptosis inhibitors ferrostatin-1 (Fer-1) and liproxstain-1 (Lip-1) treatment groups (Fig. S9D). In addition, CM NZs and HMCM treatment significantly reduced the level of lipid peroxidation compared with RSL3 treatment, and the therapeutic effect of CM NZS and HMCM treatment was about 86% of that of Fer-1 and Lip-1(Fig. S9E and S9F). These results were further confirmed that the HMCM with CM NZs as the active ingredient can inhibit ferroptosis in MCEC cells. Additionally, elevated T-GSH with concurrent MDA decrease in the CM NZs and HMCM groups (Fig. 4I and J), indicating that CM NZs as active ingredients can relieve oxidative stress. In summary, CM NZs as HMCM's core active component, inhibiting inflammatory ferroptosis by countering oxidative damage.

### 3.5 *In vivo* biosafety

Biological safety assessment is the foundation for ensuring the safe and effective application of biomaterials in medical and biological fields. These is not exhibit significant cytotoxicity of CM NZs, HM, or HMCM (0-10 μg/mL) across three cell lines (Fig. S10A–10C). Furthermore, as shown in Fig. S10D and S10E, no significant cytotoxicity was observed in the co-incubation of CM NZs, HM, and HMCM groups in RAW264.7 and MCEC cells for 12 to 24 h in the CCK-8 assay, and cell viability exceeded 95% in each group. *In vitro* evaluation confirmed that no hemolysis by CM NZs at 30 μg/mL (Fig. S11A). Subsequently, a systematic biosafety assessment of HMCM was conducted in healthy C57BL/6 mice. The results showed that progressive weight gain during oral administration cycles (Fig. S11B), and no significant tissue damage was observed in heart, liver, spleen, lung, and kidney tissue sections (Fig. S11D). Blood routine and biochemical (ALT, AST) indicators in the mice also remained within normal ranges (Figs. S12A–S12J). Lastly, elemental analysis showed preferential Cu and Mn primarily accumulated in the colon and rectum over time, without accumulating in the blood or other organs (Figs. S11C, S12K, S12M), consistent with the specific release characteristics of HMCM in the colon. Overall, HMCM exhibits exceptional biocompatibility and is negligible toxic side effects.

### 3.6 Therapeutic efficacy against DSS-induced colitis

**Fig. 5:**
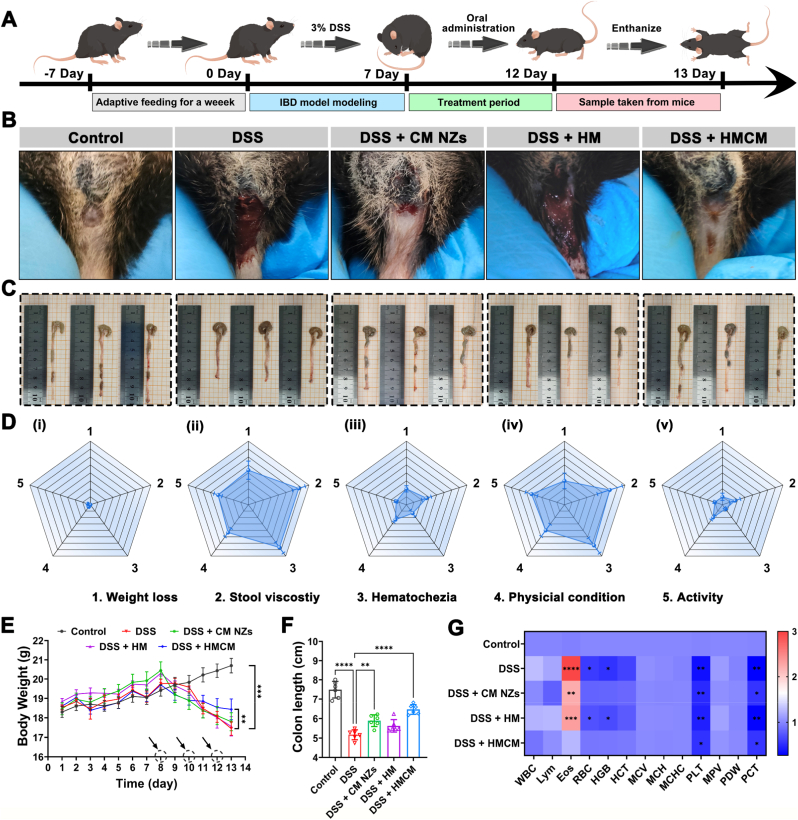
HMCM alleviated blood in stool and disease activity in DSS-induced colitis (n = 6). (A) The modeling and prevention procedures of DSS-induced colitis, (B) Photographs of the anus of mice in different treatment groups on day 13, (C) Comparison of colon length after treatment of IBD, (D) IBD disease activity index scores in different treatment groups: i) Control group; ii) DSS group; iii) DSS + CM NZs group; iv) DSS + HM group; v) DSS + HMCM group, (E) Daily weight changes in different treatment groups, (F) Quantification of colon length and (G) Heat map of blood routine indicators in different treatment groups.

Based on the experimental results, we propose an oral HMCM-targeted therapy strategy for colitis, as depicted in Fig. 5A. Mice were assigned to five treatment groups: (1) Control, (2) DSS, (3) DSS + CM NZs, (4) DSS + HM, and (5) DSS + HMCM. Compared to the DSS and HM groups, mice receiving oral CM NZs and HMCM exhibited significant improvements in weight loss, diarrhea, and hematochezia severity, with Disease Activity Index (DAI) scores nearly matching those of the healthy control group (Fig. 5B, D, 5E, and Fig. S13). The HMCM group demonstrated the most substantial recovery effects. Additionally, the colon length in the HMCM group was significantly greater than in the other groups (Fig. 5C and F). The results of blood and blood biochemical indexes showed that compared with the Control group, the DSS group had significant changes in inflammatory indicators (HGB, RBC, Eos, etc.) and an increase in ALT. However, there was no significant difference in the values of each index between the HMCM group and the Control group (Fig. 5G and Fig. S14). In addition, we used 5-aminosalicylic acid (5-ASA), a first-line drug for the treatment of IBD, for comparative experiments. As shown as Figs. S15 and S16, HMCM and 5-ASA significantly alleviated DSS-induced colitis compared with the model group. Surprisingly, DAI score, recovery of colon length, and integrity of colonic epithelial structure demonstrated that HMCM was superior to 5-ASA. The above findings initially confirm the clinical potential of oral HMCM for the treatment of IBD.

**Fig. 6:**
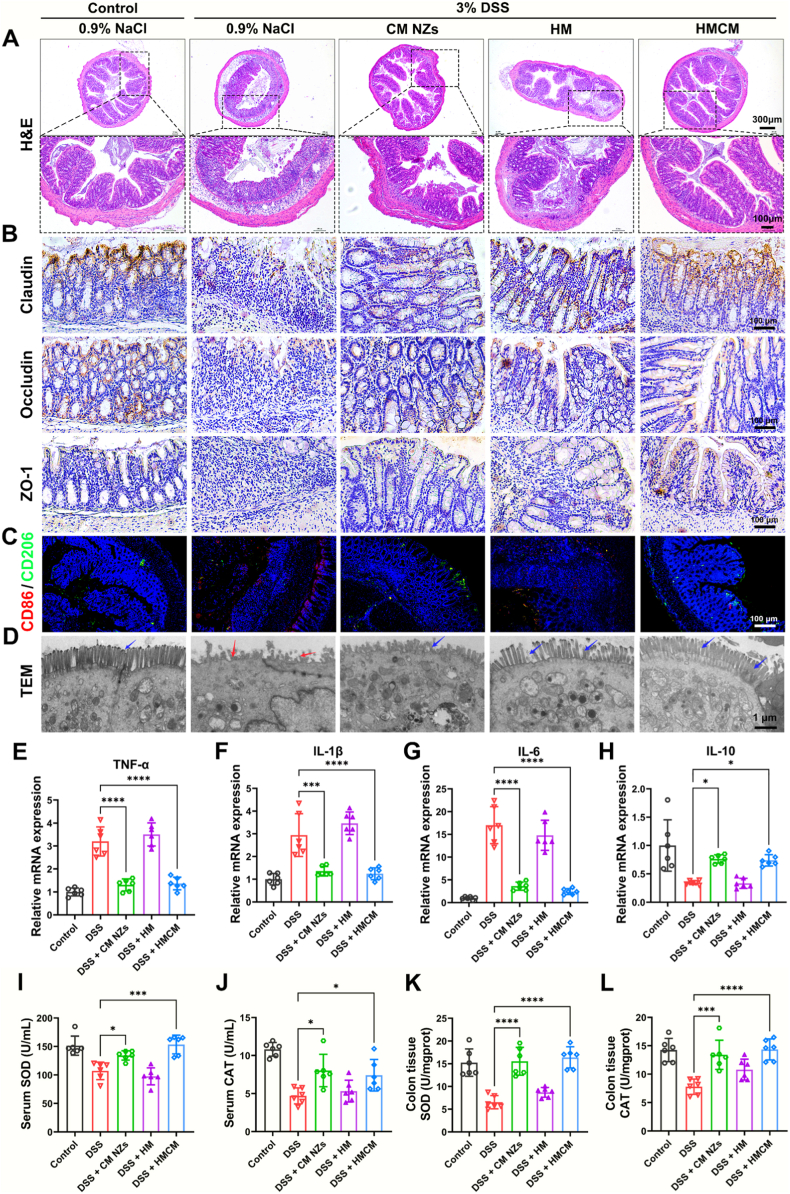
Treatment of HMCM in a DSS-induced colitis mouse model. (A) H&E staining, (B) IHC staining of Claudin, ZO-1 and Occludin, (C) IF staining of CD86/CD206, (D) TEM images of different treatment groups, (E) TNF-α, (F) IL-1β, (G) IL-6, and (H) IL-10 in different treatment groups, (I) SOD and (J) CAT enzyme activities in serum from different treatment groups, (K) SOD and (L) CAT enzyme activities in colon tissue from different treatment groups.

**Fig. 7:**
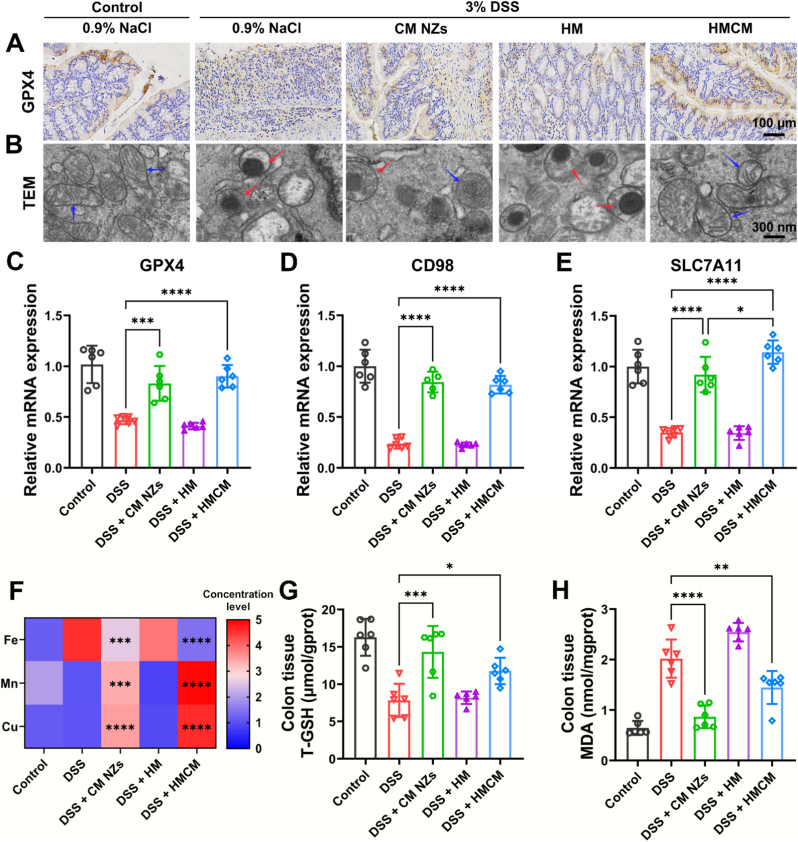
HMCM reduces the sensitivity of intestinal epithelial cells to ferroptosis at sites of inflammation. (A) Colon tissue sections labeled with GPX4 antibody. (B) mitochondrial TEM images (Red arrow: damaged mitochondria, Blue arrow: normal mitochondria). Expression of ferroptosis genes in different treatment groups: (C) GPX4 mRNA expression, (D) CD98 mRNA expression, and (E) SLC7A11 mRNA expression. (F) Fe, Mn, and Cu content in colon tissue from different treatment groups (Fe: 50 μg/g = 1 level, Mn: 6 μg/g = 1 level, Cu: 3 μg/g = 1 level). (G) T-GSH and (H) MDA in colon tissue from different treatment groups.

This study assessed intestinal tissue pathology and tight junction protein expression (Claudin, Occludin, and ZO-1) across treatment groups. H&E staining (Fig. 6A) and TEM (Fig. 6D) revealed that HMCM treatment preserved colonic epithelial structure integrity. The intestinal barrier serves as a critical defense line in the digestive system, acting like a meticulously constructed “wall” that effectively separates the internal environment of the intestine from the body's internal environment [62]. Claudin, Occludin, and ZO-1 are core proteins that constitute the intestinal tight junctions and are closely associated with the integrity of the intestinal barrier [63,64]. IHC results demonstrated superior restoration of damaged intestinal barriers in the HMCM group (Fig. 6B), attributable to HM's targeted adhesion to inflammatory sites and CM NZs' excellent reactive oxygen species scavenging capacity. Colon copper/manganese distribution (Fig. 7F) further confirmed HMCM's targeted delivery and efficient utilization. The polarization state of macrophages is a key factor in improving the severity of inflammatory bowel disease and tissue repair capacity. While *M1* macrophages exacerbate inflammation, *M2* phenotypes facilitate resolution and repair [65]. Immunofluorescence staining revealed HMCM treatment shifted colonic macrophages from pro-inflammatory *M1* (CD86^+^, red) to reparative *M2* (CD206^+^, green) phenotypes (Fig. 6C). Compared with the DSS group, the green fluorescence in the HMCM group was increased by 2.9 times, and the red fluorescence was decreased by 3.8 times (Fig. S17), this result suggests that HMCM alleviates colitis by balancing *M1*/*M2* macrophage polarization. Additionally, HMCM significantly suppressed pro-inflammatory cytokines (TNF-α, IL-1β, IL-6) and promoted anti-inflammatory IL-10 expression (Fig. 6E–H). Furthermore, HMCM significantly increased CAT and SOD enzymes activity in serum and colon tissue (Fig. 6I–L). In summary, the results indicate that HMCM effectively treats inflammatory bowel disease through multiple mechanisms, including repairing the intestinal barrier, regulating macrophage polarization, balancing inflammatory cytokines, and enhancing antioxidant defense capabilities.

### 3.7 *In vivo* anti-ferroptotic activity of HMCM

We next elucidated HMCM's anti-ferroptotic mechanism. There are reports that prove GPX4 and SLC7A11 as central ferroptosis regulators [66]. Downregulation of GPX4 impairs lipid ROS scavenging and promotes ferroptosis [67]. IHC revealed significantly upregulated GPX4 expression in HMCM-treated mice (Fig. 7A). qRT-PCR demonstrated HMCM enhances *GPX4* expression via *CD98* and *SLC7A11* upregulation (Fig. 7C–E), confirming protection against GPX4-mediated ferroptosis. TEM images of colon tissue showed that mitochondria in the HMCM-treated group avoided damage caused by ferroptosis, retaining a structure and morphology nearly identical to that of healthy mice (Fig. 7B). Given that free iron accumulates extensively during ferroptosis, we measured total iron content in colon tissue using ICP-MS. ICP-MS analysis showed HMCM significantly reduced total colonic iron content versus all treatments (Fig. 7F). Concomitantly, HMCM elevated T-GSH while reducing MDA levels (Fig. 7G and H), indicating oxidative stress mitigation and thereby inhibited ferroptosis.

### 3.8 Regulation of intestinal microbiota

**Fig. 8:**
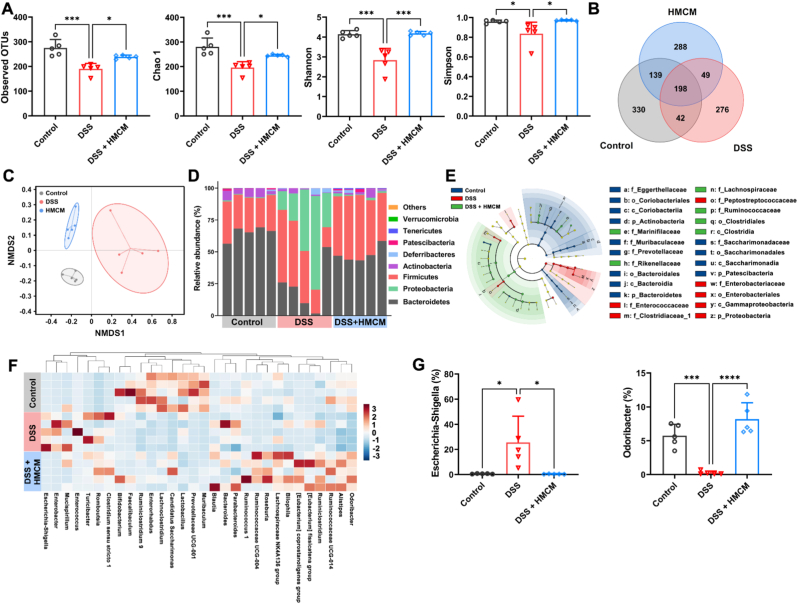
HMCM regulates intestinal microbiota imbalance and improves the intestinal microenvironment. (A) The α diversity index of observed species, Chao1, Shannon and Simpson. (B) Venn diagram of species among the various groups. (C) NMDS score plot of β diversity of the gut microbiome. (D) Histogram of abundance between groups. (E) LEfSe analysis of differential species ring diagram. (F) Heatmap at genus level. (G) Genus abundance of Escherichia-Shigella and Odoribacter after different treatment groups.

The pathogenesis of IBD is closely associated with an imbalance in the gut microbiota [68]. To investigate the regulatory effects of HMCM on the gut microbiota, fecal samples were collected and subjected to 16S rRNA gene amplicon sequencing to analyze the composition of the gut microbiota. Alpha diversity analysis revealed significant differences in gut microbial diversity and abundance between DSS-induced colitis mice and the normal and HMCM groups, as confirmed by quantitative species statistics, Shannon index, and Chao1 index (Fig. 8A). Venn diagrams were constructed to illustrate the shared and unique taxonomic units across the three groups (Fig. 8B). Additionally, non-metric multidimensional scaling (NMDS) plots showed that the microbial community structures of DSS-induced colitis mice and healthy mice were distinctly separated. However, the intestinal microbial community structure of the HMCM treatment group was more similar to that of normal mice (Fig. 8C). Subsequently, we conducted an in-depth analysis of the gut microbiota. The composition of the gut microbiota was analyzed at the phylum and genus levels. Based on phylum-level abundance, the microbial composition of the normal group and HMCM group showed high similarity (Fig. 8D–F).

Quantitative analysis indicated that HMCM effectively increased the ratio of *Bacteroidetes* to *Firmicutes* in intestinal bacteria. As one of the major identified bacterial phyla, *Proteobacteria* is closely associated with the pathogenesis and progression of IBD [10,69]. HMCM effectively suppressed the abundance of Proteobacteria in the colon. *Actinobacteriota* are beneficial intestinal bacteria [70]. Compared to DSS-induced colitis mice, mice treated with HMCM exhibited a significant increase in the abundance of *Actinobacteriota*. At the genus level, HMCM also significantly increased the relative abundance of *Odoribacter* (beneficial bacteria) [71]and reduced that of *Escherichia-Shigella* (pathogenic bacteria) [72] (Fig. 8G). These results confirm that HMCM effectively reverses DSS-induced dysbiosis and restores intestinal microbial balance.

## 4 Conclusions

In summary, we develop a colon-targeted composite, hydrogel microspheres@Cu-Mn_3_O_4_ NZs (HMCM), for oral IBD treatment by encapsulating Cu-Mn_3_O_4_ nanozymes (CM NZs) in calcium alginate microspheres (HM). The mixed-valence Mn^2+^/Mn^3+^ and Cu^+^/Cu^2+^ in CM NZs confer excellent SOD-, CAT-, and GPx-mimetic activities, enabling potent broad-spectrum ROS scavenging. HMCM shows specific dissolution in the colonic microenvironment, ensuring targeted delivery to inflammatory sites while avoiding gastric degradation. In DSS-induced colitis, HMCM acts through three synergistic mechanisms: i) enhancing anti-inflammatory and antioxidant responses by reducing oxidative stress, promoting *M1*-to-*M2* macrophage polarization, and modulating cytokine levels; ii) ameliorating intestinal injury by inhibiting ferroptosis via GPX4/SLC7A11 upregulation and repairing tight junctions; and iii) reshaping gut microbiota homeostasis. Additionally, HMCM possesses favorable biocompatibility and highly specific retention in the colon. Overall, HMCM demonstrates robust efficacy *in vitro* and *in vivo*, presenting an efficient targeted strategy for IBD.

## Funding

This work was supported by the National 10.13039/501100018542Natural Science Foundation of Sichuan Province (Grant No. 24NSFSC0279) and Joint Funds of the 10.13039/501100001809National Natural Science Foundation of China (Grant No. U22A20513).

## CRediT authorship contribution statement

**Wei Fan:** Data curation, Software, Writing – original draft, Writing – review & editing. **Yinyin Chen:** Investigation. **Wenshuang Chen:** Data curation. **Zisong Gao:** Investigation. **Zhongke Yang:** Formal analysis. **Hongyan Li:** Formal analysis. **Aimin Wu:** Project administration. **Xianxiang Wang:** Methodology, Project administration.

## Declaration of competing interest

The authors declare that they have no known competing financial interests or personal relationships that could have appeared to influence the work reported in this paper.

## Data Availability

Data will be made available on request.
