# Supplementary material for: Multifunctional Cu-doped Mn3O4 nanozyme hydrogel microspheres for oral targeted treatment of inflammatory bowel disease
Source: Mater Today Bio. 2026 Feb 13;37:102932. doi: 10.1016/j.mtbio.2026.102932 (PMC12924754; doi:10.1016/j.mtbio.2026.102932)
Supplement: Multimedia component 1 [file mmc1.docx]

**Multifunctional Cu-doped Mn_3_O_4_ nanozyme hydrogel microspheres for oral targeted treatment of inflammatory bowel disease**

Wei Fan^a^, Yinyin Chen^b^, Wenshuang Chen^a^, Zisong Gao^c^, Zhongke Yang^a^, Hongyan Li^d^, AiminWu ^e*^, Xianxiang Wang^a*^

^a^*College of Science, Sichuan Agricultural University, Chengdu 611130, Sichuan, China*

*^b^College of Agronomy, Sichuan Agricultural University, Chengdu 611130, Sichuan, China*

*^c^Veterinary Pharmacy in College of Animal Science and Technology of Jiangxi Agricultural University, Nanchang 330045, JiangXi, China*

*^d^Dafeng Street Taiping Community Health Service Center, Chengdu 610504 Sichuan, China*

^e^*Institute of Animal Nutrition, Sichuan Agricultural University, Chengdu 611130, China*

*****To whom correspondence should be addressed: Aimin Wu and Xianxiang Wang

E-mail address: wuaimin0608@163.com (Aimin Wu), xianxiangwang@hotmail.com (Xianxiang Wang).

**Materials and methods**

*Materials and instruments.*

All chemicals used in the experiment were of analytical grade and could be used directly. According to standard procedures, double-distilled water was used to prepare acetic acid-sodium acetate buffer solution (0.2 M) and phosphate buffer solution (0.01 M). copper acetate (Cu(Ac)_2_), manganese acetate (Mn(Ac)_2_), reduced glutathione (GSH), pluronic F-127, hyaluronic acid (MW: 100,000-200,000), sodium alginate, 5,5'-dithiobis (2-nitrobenzoic acid) (DTNB), 3,3',5,5'-tetramethylbenzidine (TMB), and nitrotetrazolium blue chloride (NBT) were purchased from Shanghai Macklin Biochemical Co., Ltd. Anhydrous calcium chloride (CaCl_2_), 30% H_2_O_2_ were purchased from Chengdu Kelong Chemical Co., Ltd. The malondialdehyde detection kit, glutathione detection kit, and other detection kits were purchased from NanJing JianCheng Bioengineering Institute. 2,7-dichlorofluorescein diacetate (DCFH-DA), C11-BODIPY, Far-Red Labile Fe^2+^ probe, CCK-8, and bicinchoninic acid (BCA) protein assay kits were used.

Characterization parameters (morphology, size, etc.) of the corresponding materials were obtained using high-resolution transmission electron microscopy (HRTEM, FEI Tecnai G2 F20, USA) and scanning electron microscopy (SEM, Gemini SEM 300, Germany). XPS spectra were measured using an ESCALAB 250Xi photoelectron spectrometer (Thermo Scientific, USA) to determine the elemental composition. FTIR spectra were obtained using a Fourier transform infrared spectrometer (FTIR-8400S, Shimadzu, Japan) to identify functional groups. The crystal structure of the products was characterized using an X-ray diffractometer (XRD, DX2700, Dandong, China). The Zeta potential was measured using the NANO ZS instrument, and the scavenging of free radicals was analyzed using electron paramagnetic resonance (EPR, Bruker A300). The content of relevant metal elements was tested using an inductively coupled plasma emission spectrometer (ICP-MS, NexlON 2000, USA). Fluorescence and ultraviolet-visible spectra were measured using a fluorescence spectrophotometer (Hitachi, F-4500, Japan) and an ultraviolet-visible spectrophotometer (AOE instruments, A390, Shanghai, China), respectively.


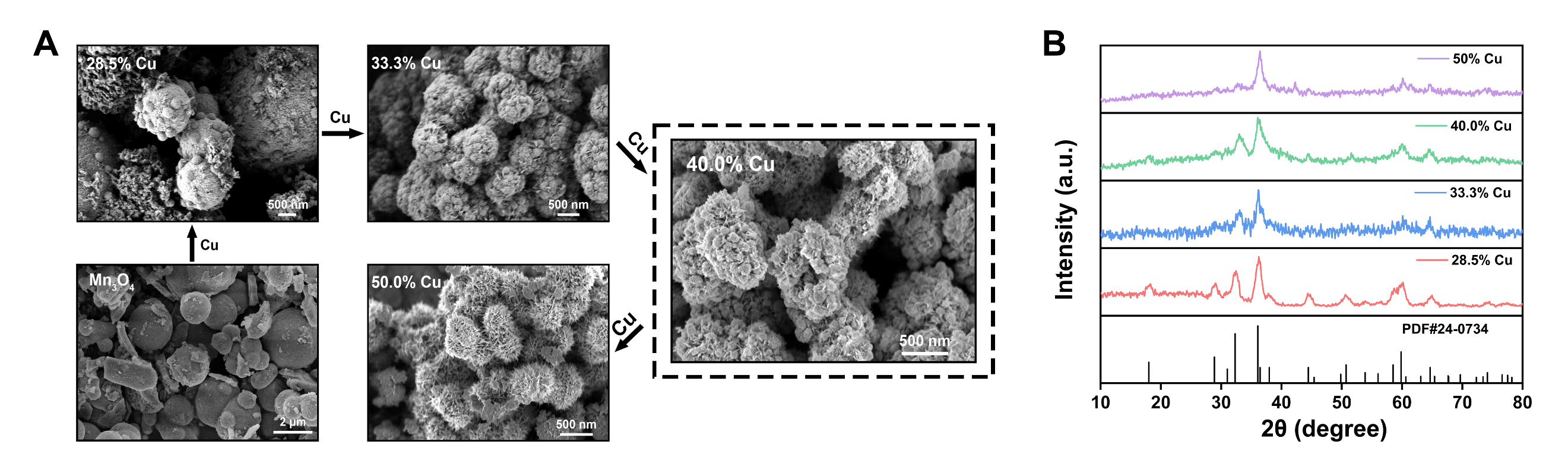


**Fig. S1.** (A) SEM images of CM NZs synthesized with different copper content doping. (B) XRD patterns of CM NZs with different copper doping concentrations


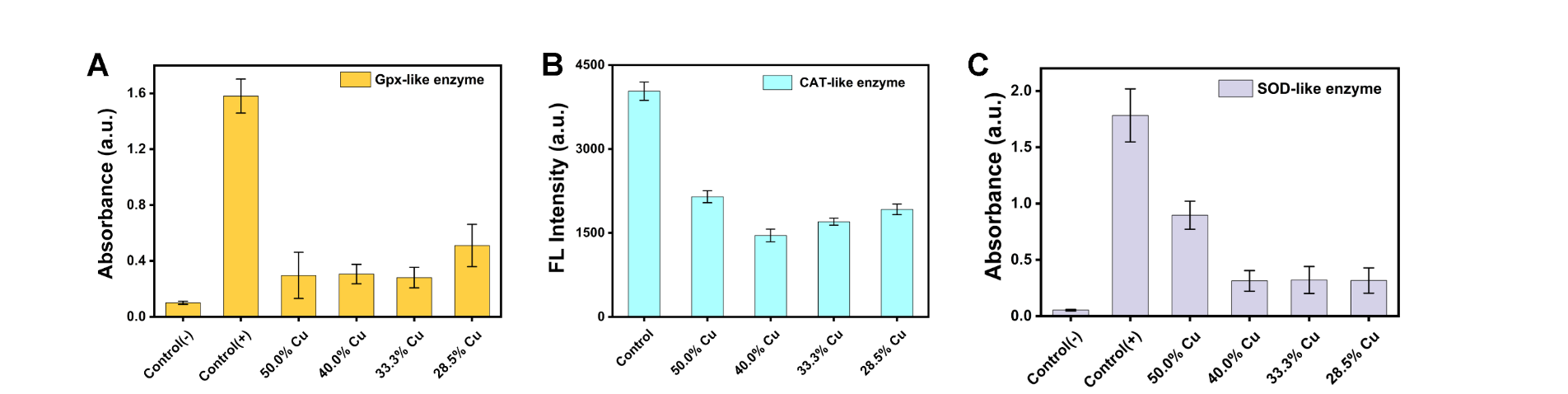


**Fig. S2.** (A) GPx, (B) CAT, and (C) SOD enzyme activities of CM NZs with different copper doping concentrations


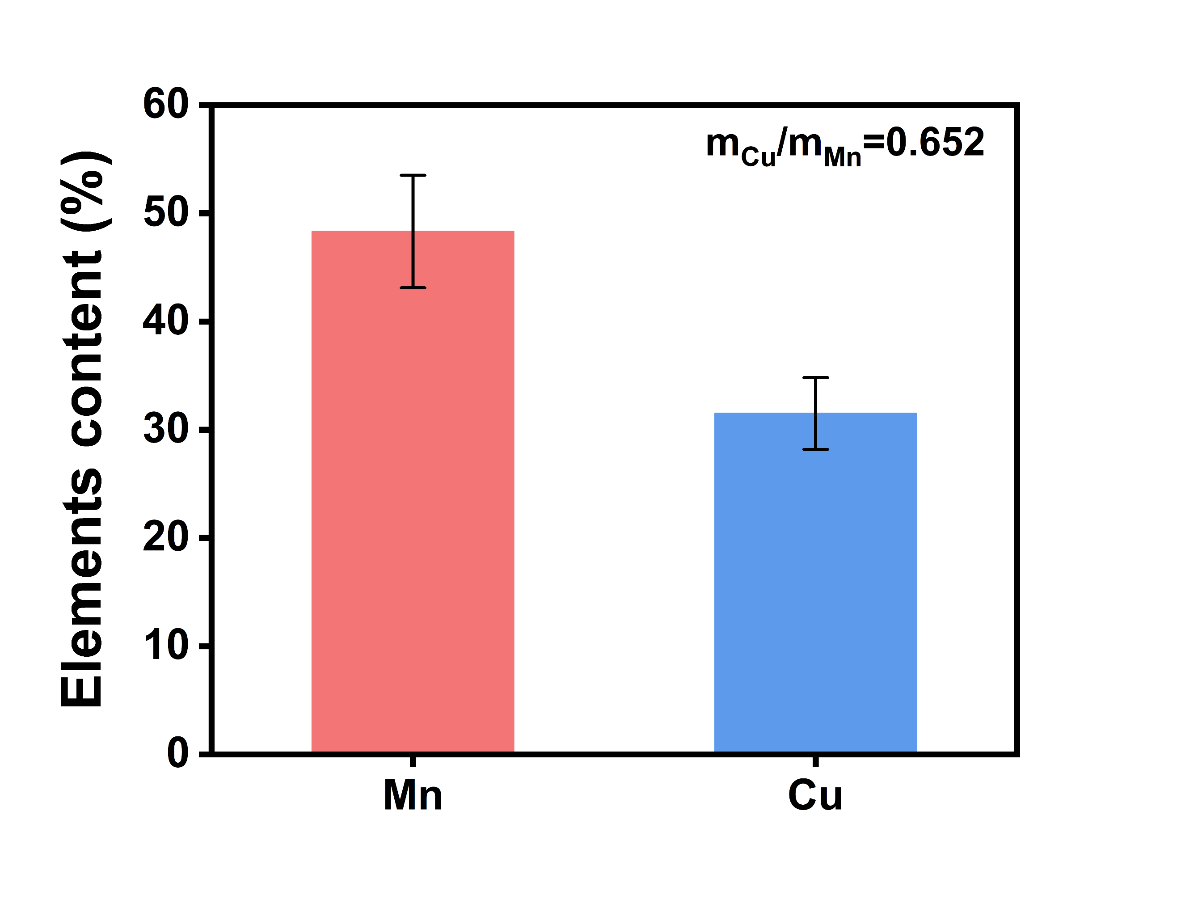


**Fig. S3.** Copper and manganese content in CM NZs


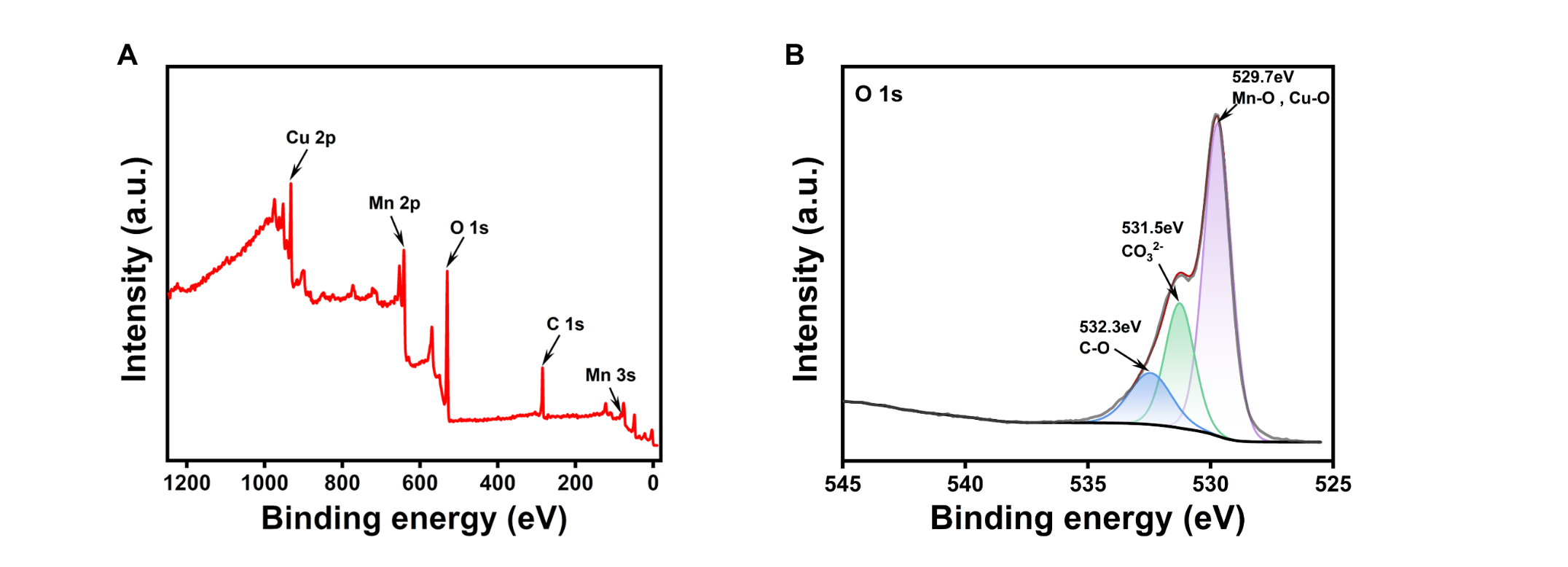


**Fig. S4.** (A) Survey XPS spectra of CM NZs. (B) XPS fine spectrum of O1s


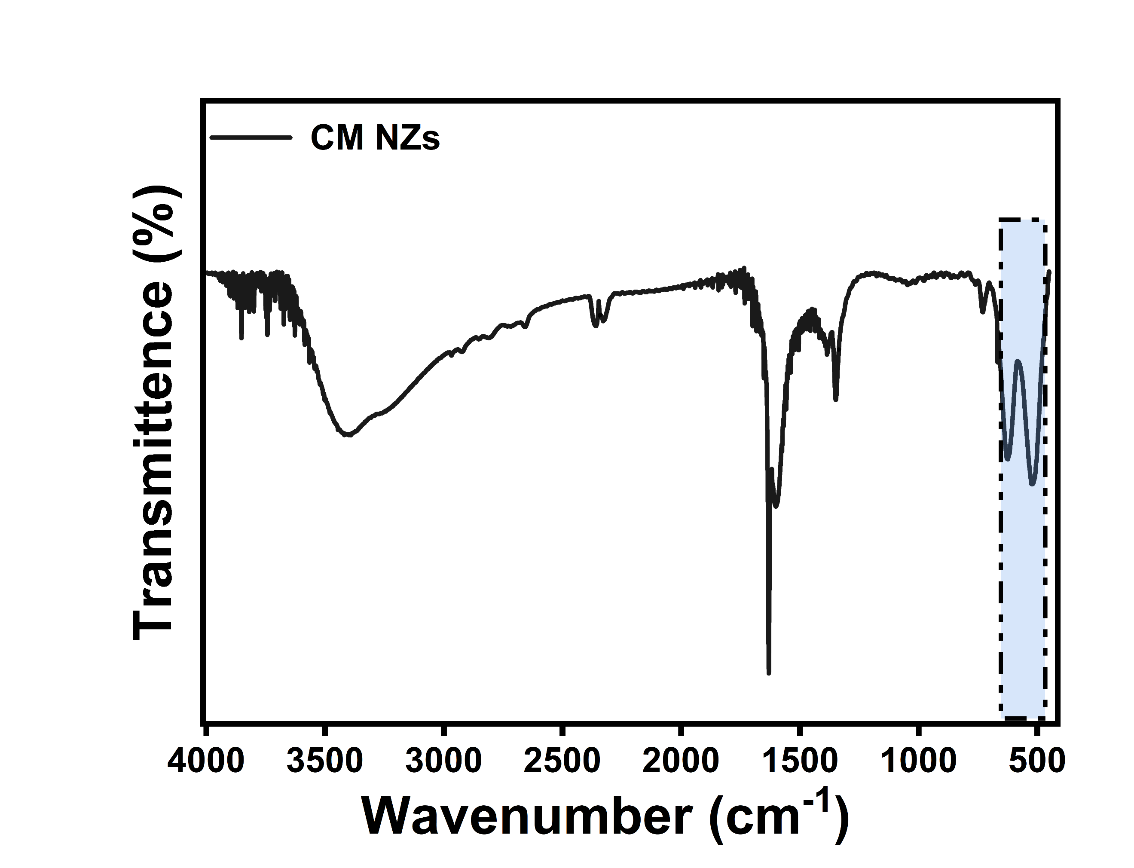


**Fig. S5.** FTIR spectrum of CM NZs


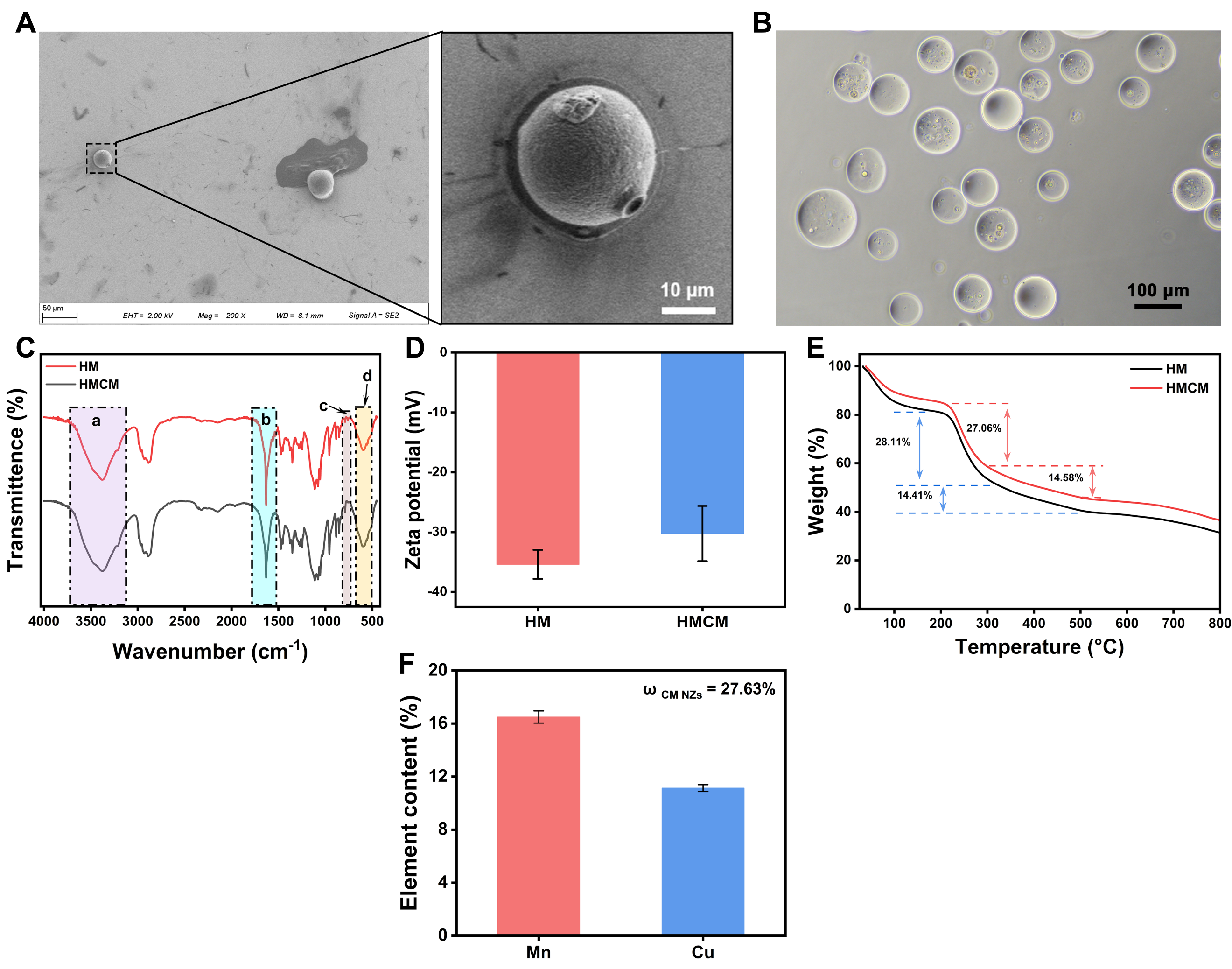


**Fig. S6.** (A) SEM image of hydrogel microspheres. (B) Microscopic image of hydrogel microspheres. (C) FTIR spectra of HM and HMCM. (D) Zeta Potential of HM and HMCM. (E) Thermogravimetric analysis of HM and HMCM. (F) Cu and Mn content in HMCM.


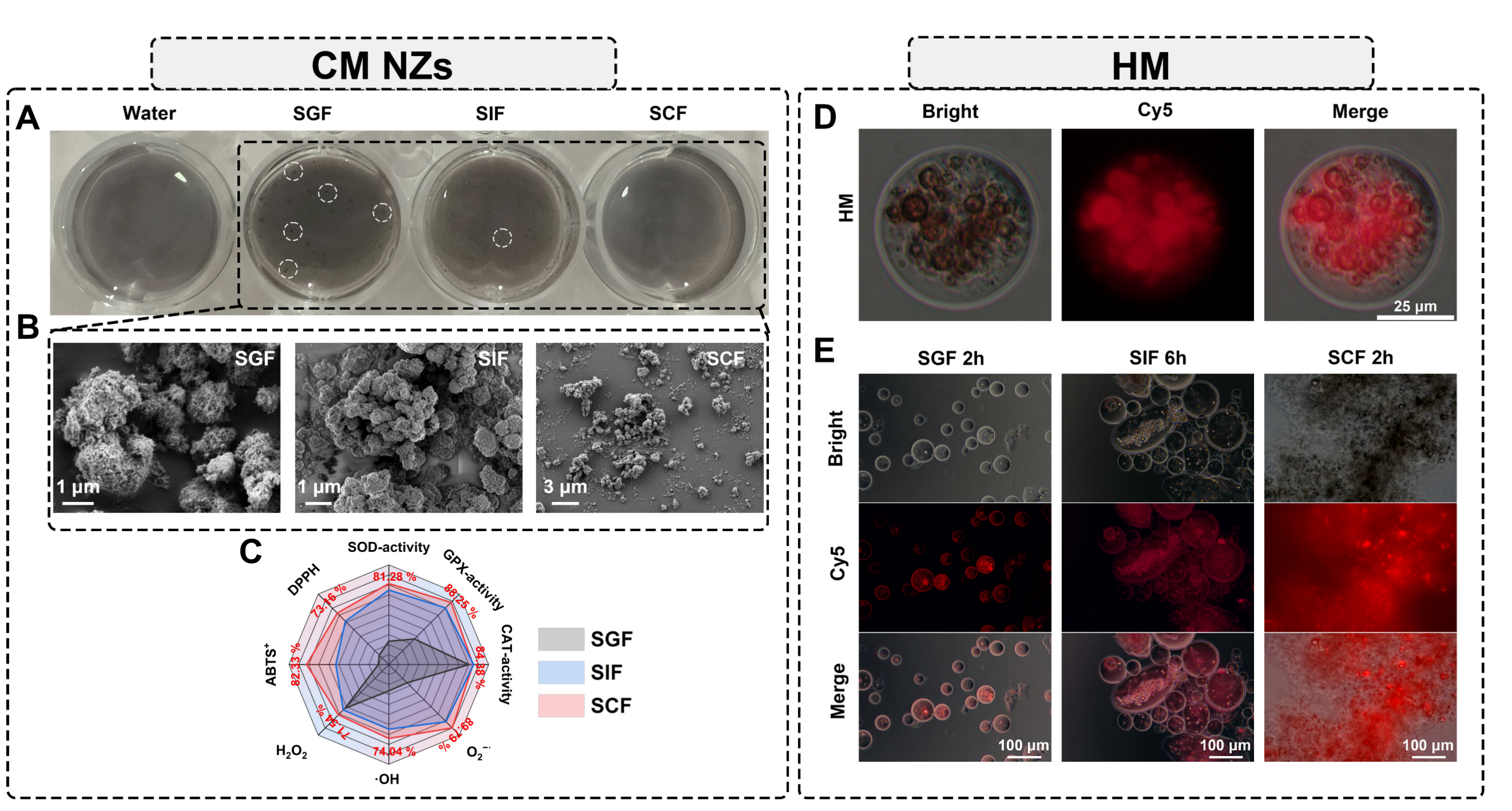


**Fig. S7.** (A) Images of CM NZs dispersed in solutions of water, SGF, SIF and SCF. (B) SEM images of CM NZs treated with SGF, SIF and SCF. (C) Radar plot of enzyme activity and free radical scavenging ability of CM NZs after treatment with SGF, SIF and SCF. (D) Cy5-labeled HM. (E) Microscopic images showing morphological changes in Cy5-labeled HM after treatment with simulated gastrointestinal fluid.


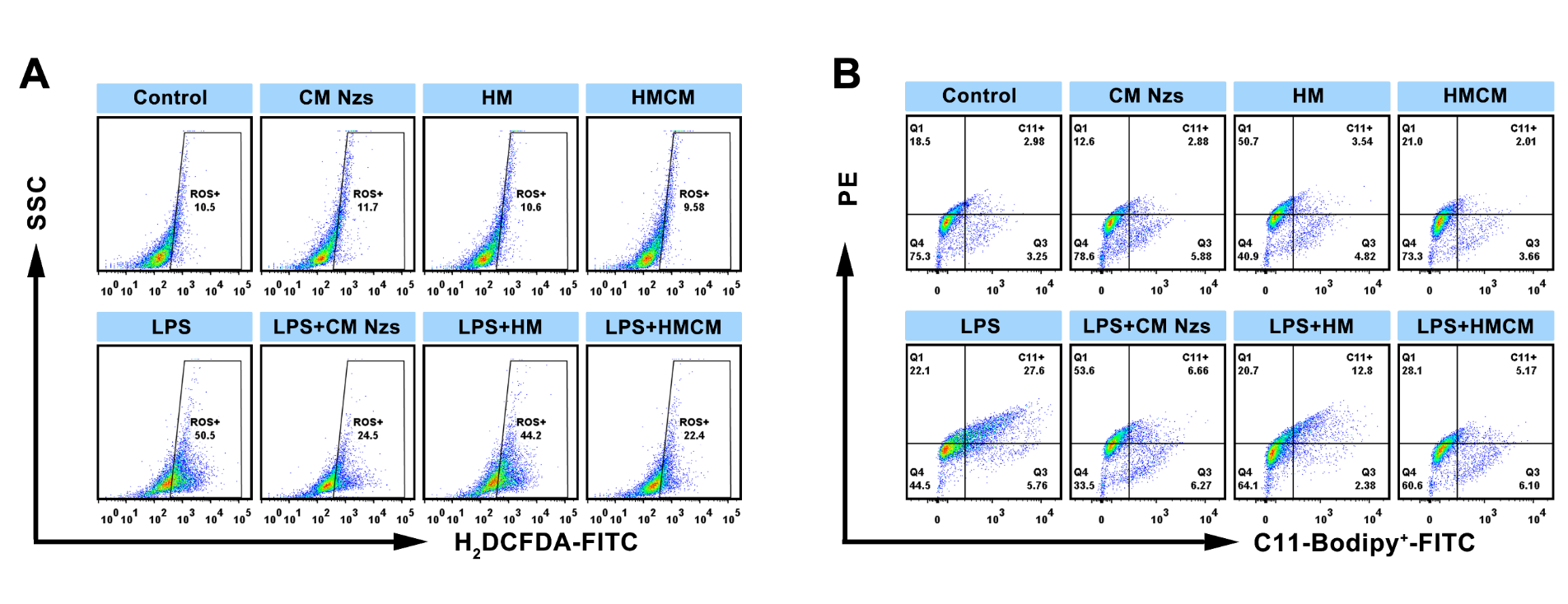


**Fig. S8.** (A) FACS analysis of ROS content in RAW 264.7 cells treated with different experimental groups (n=4). (B) FACS analysis of lipid ROS content in RAW 264.7 cells treated with different experimental groups (n=4).


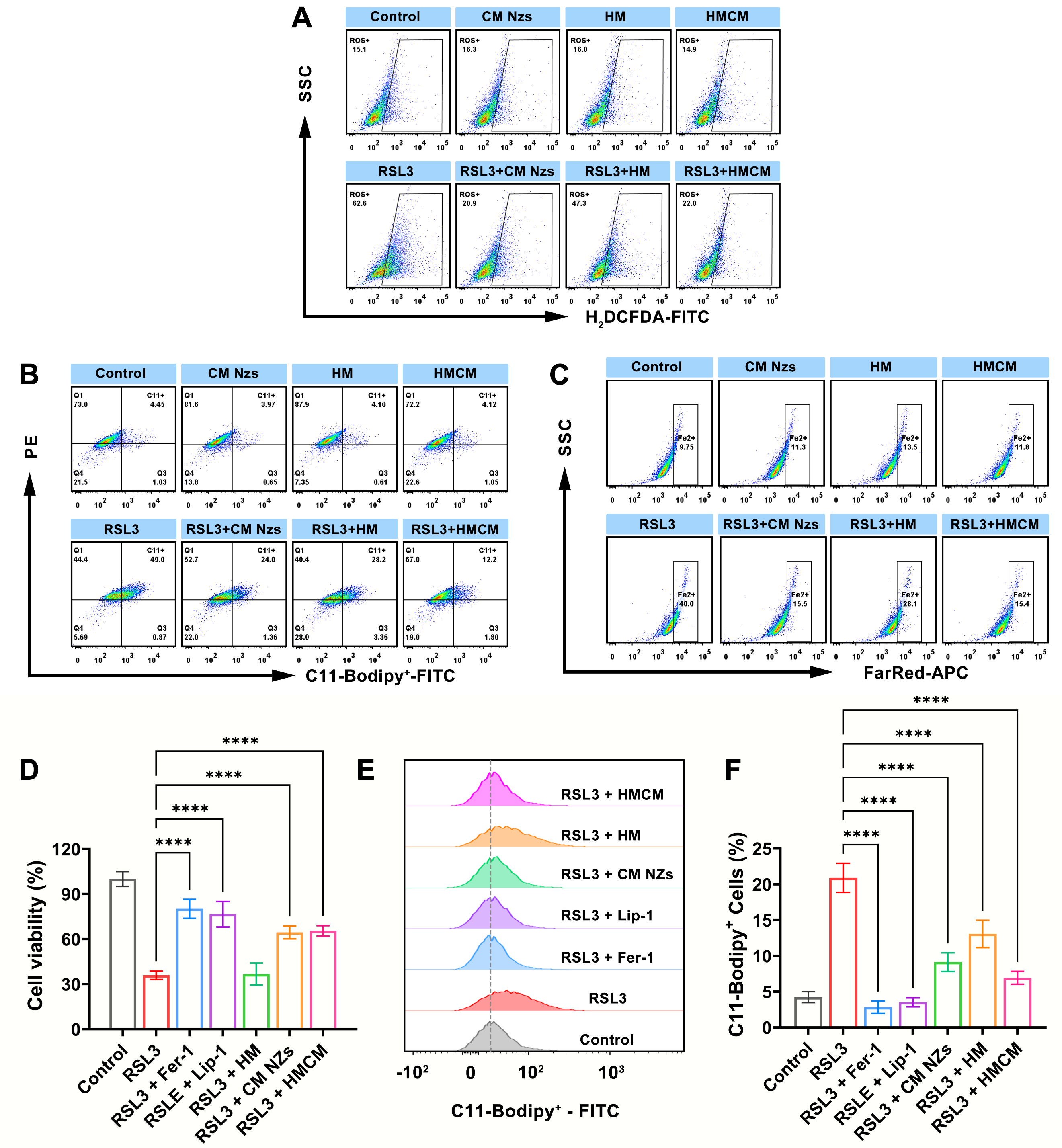


**Fig. S9.** (A) FACS analysis of ROS content in MCEC cells treated with different experimental groups (n=4). (B) FACS analysis of lipid ROS content in MCEC cells treated with different experimental groups (n=4). (C) FACS analysis of Fe^2+^ content in MCEC cells treated with different experimental groups (n=4). (D) CCK-8 assays of MCEC cells treated with Fer-1, Lip-1, CM NZs, HM and HMCM, respectively. (E-F) Lipid ROS in MCEC cells from different treatment groups were measured by flow cytometry.


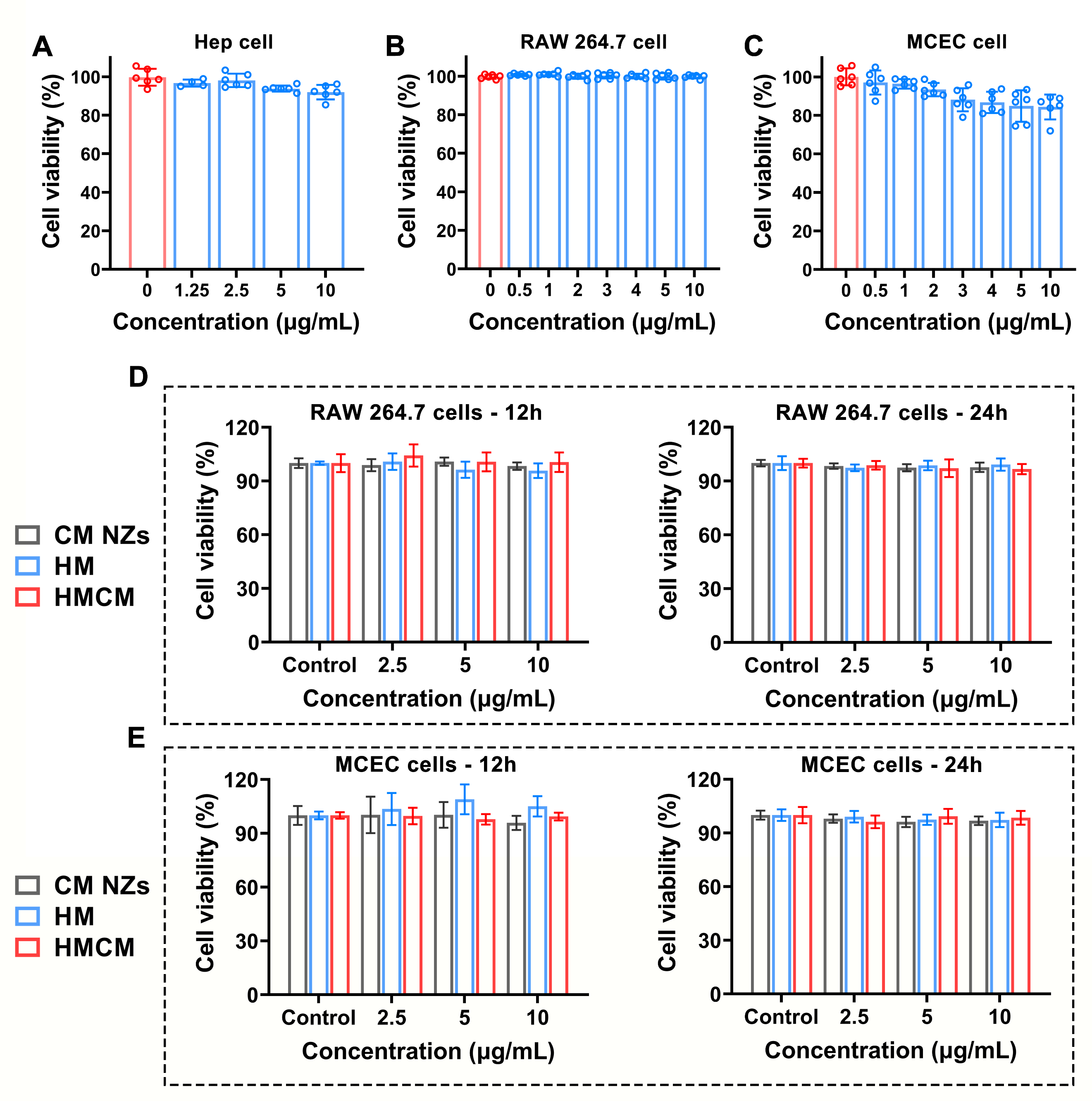


**Fig. S10.** Assay of viability of (A) Hep cells, (B) RAW264.7 cells, and (C) MCEC cells after treatment with CM NZs. Viability assay of (D) RAW264.7 and (E) MCEC cells after CM NZs, HM or HMCM treatment with 12h and 24h.


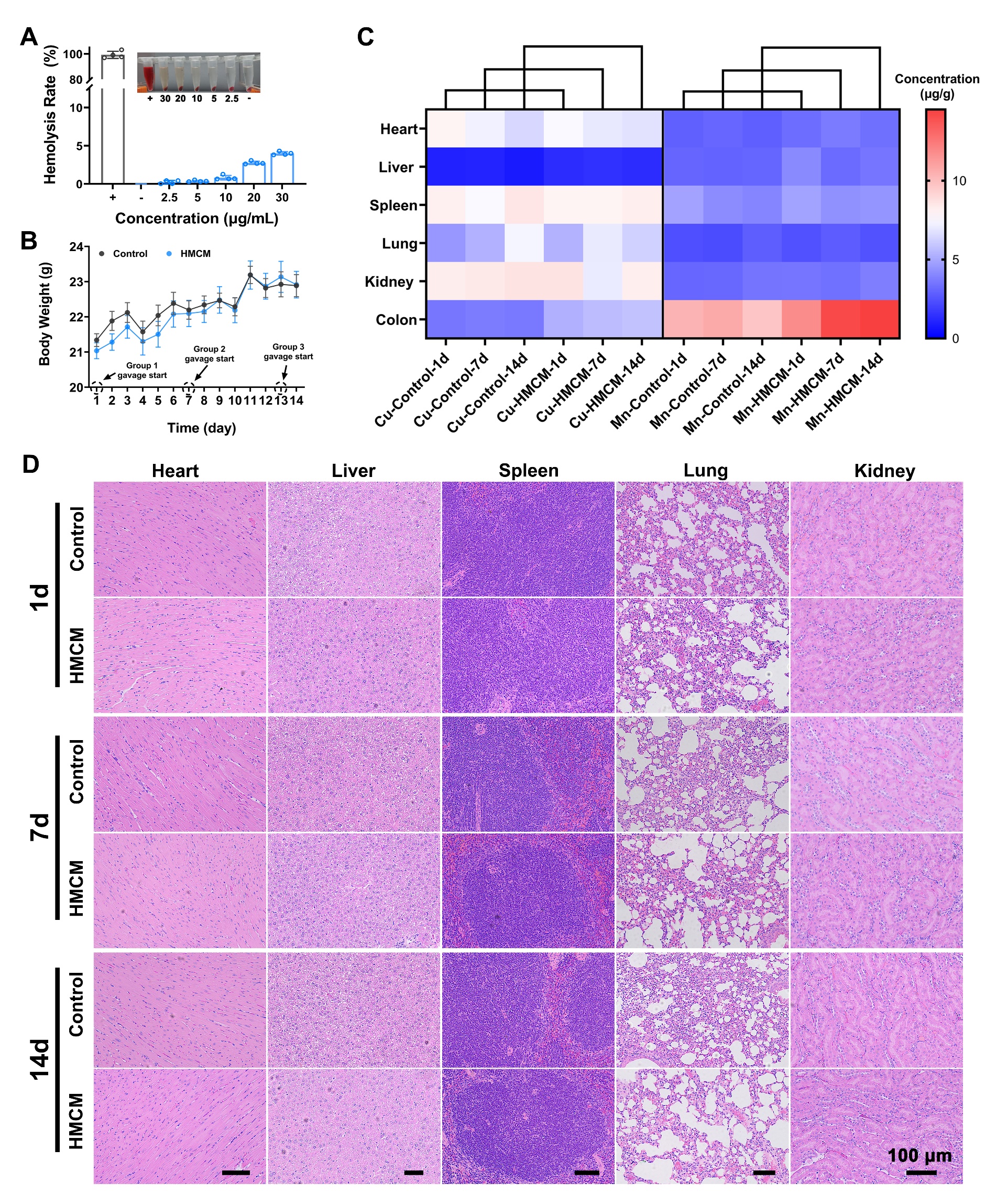


**Fig. S11.** **Biosafety experiments of HMCM.**

(A) Hemolytic testing of CM NZs (n=4). (B) Weight gain curves of mice in different groups during the experiment (n=5). (C) Cu and Mn content in major organs and intestinal tissues of mice in different treatment groups at 1, 7, and 14 days after feeding (n=5). (D) H&E section images of major organs in mice from different treatment groups at 1, 7, and 14 days after feeding.


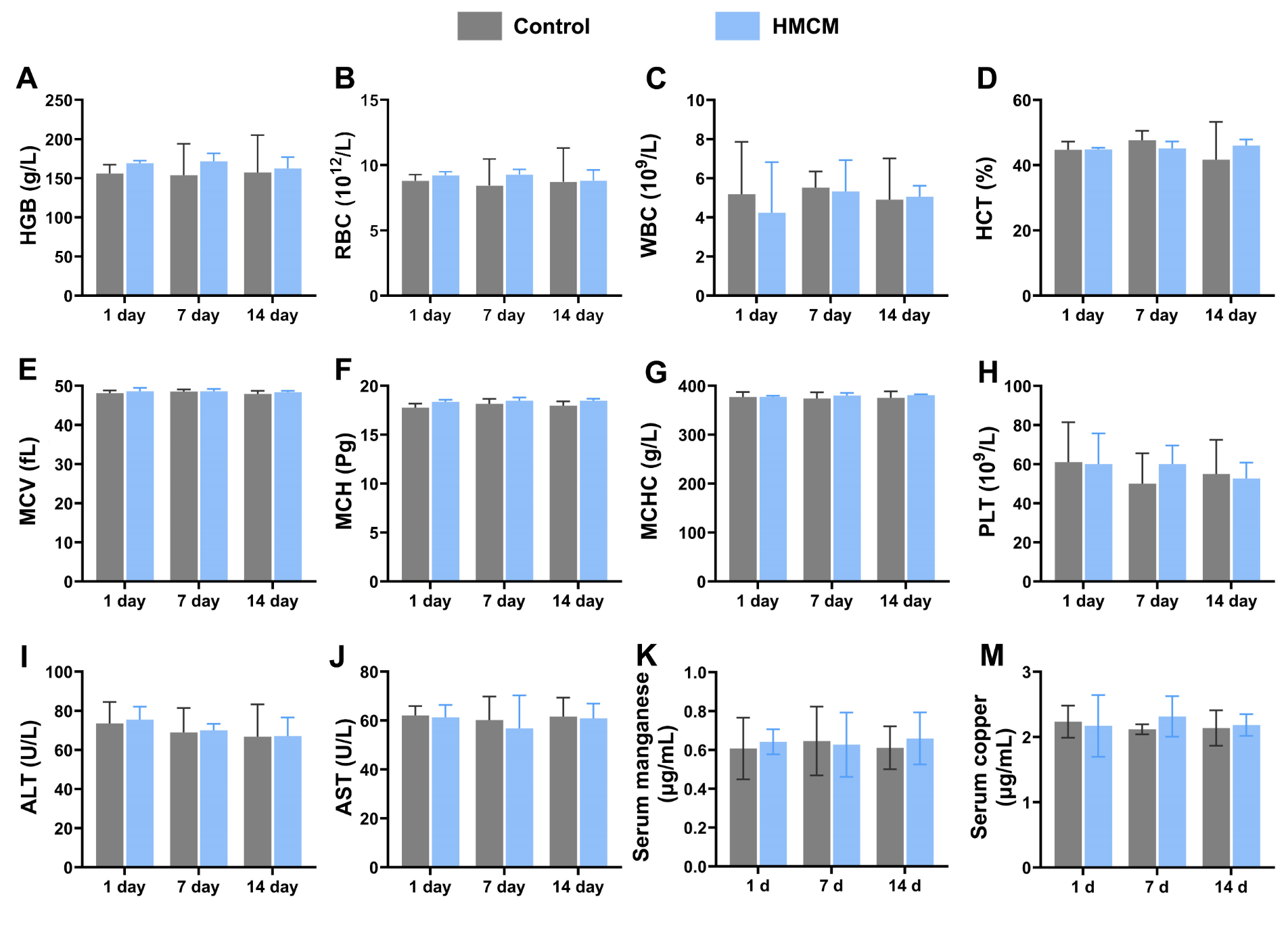


**Fig. S12. Bar charts showing the main indicators of routine blood tests in different groups of mice in the 1day, 7day, and 14-day biosafety experiments.**

(A) HGB. (B) RBC. (C) WBC. (D) HCT. (E)MCV. (F) MCH. (G) MCHC. (H)PLT. (I) Alanine aminotransferase (ALT). (J) Aspartate aminotransferase (AST). (K) Serum manganese. (M) Serum copper.


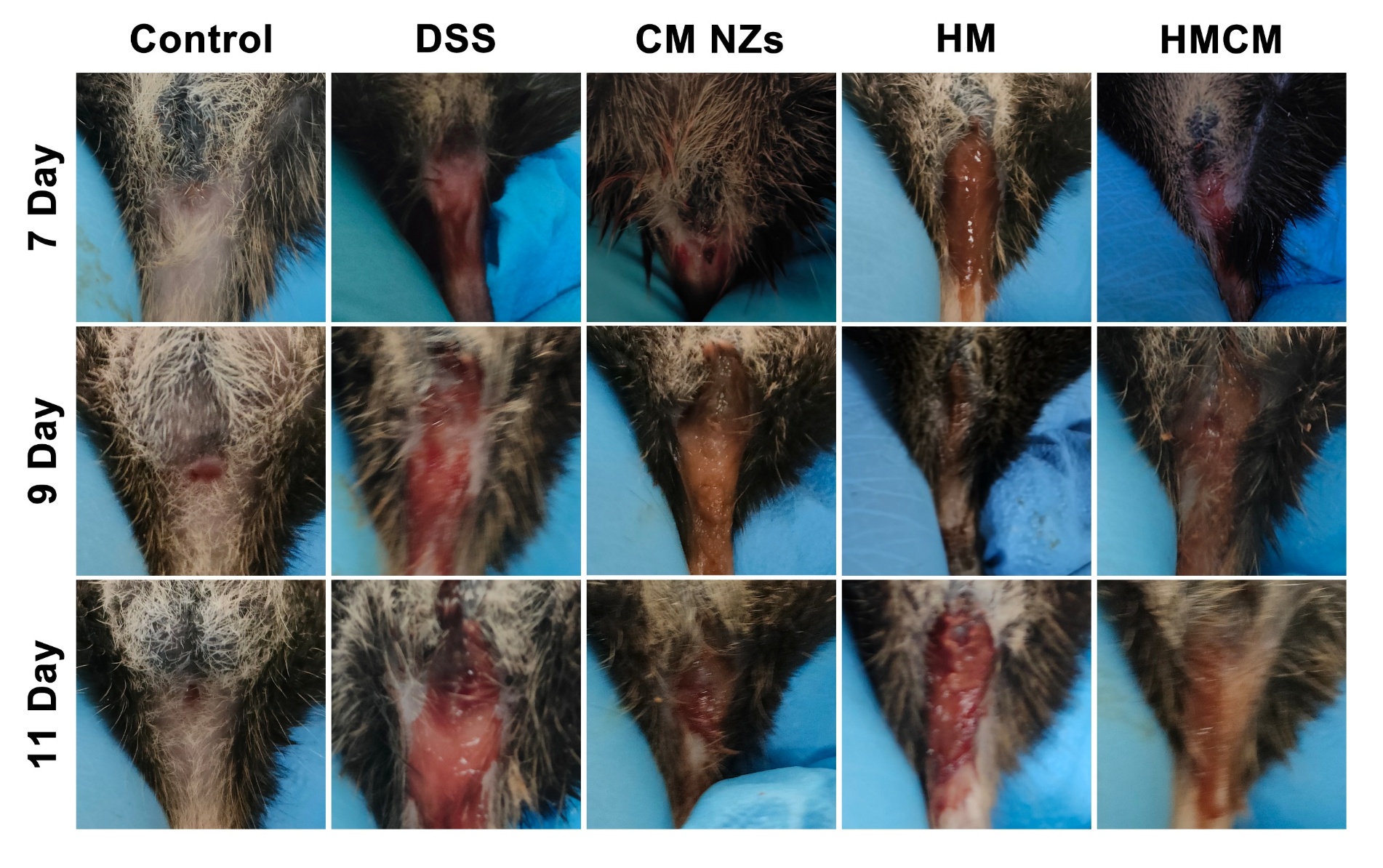


**Fig. S13.** Photographs of the anus of mice in different treatment groups on day 7, 9, 11.


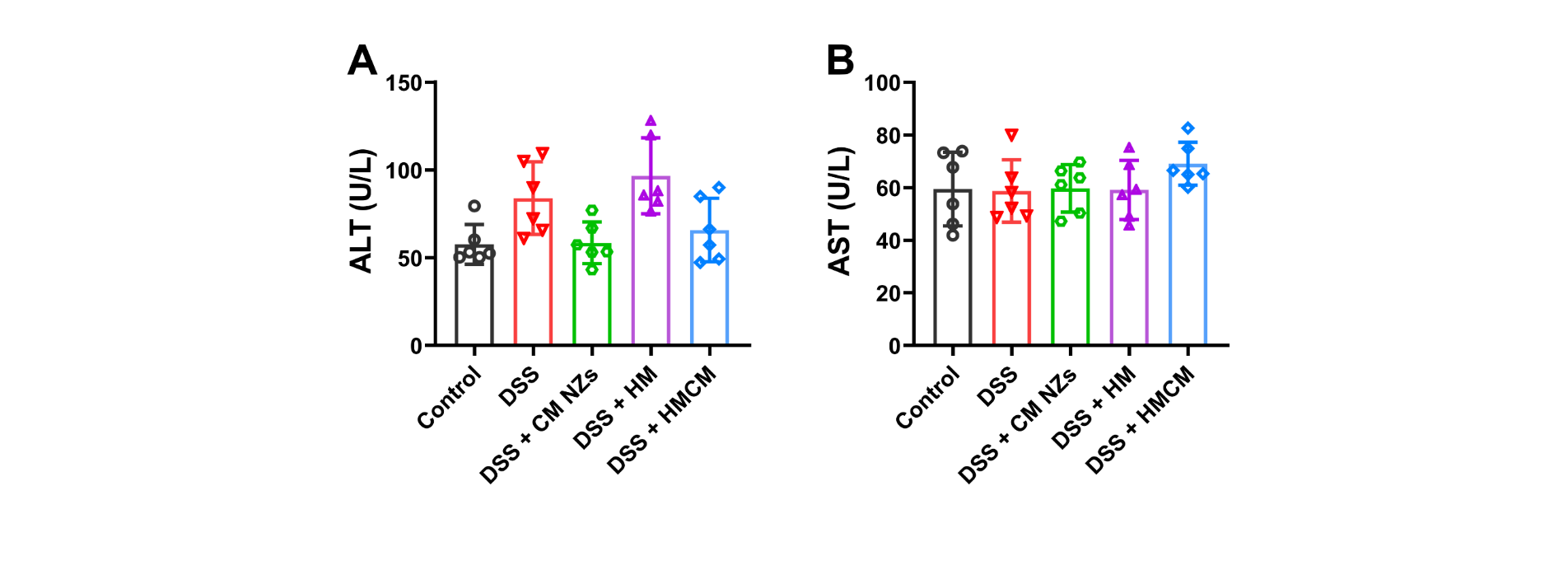


**Fig. S14.** Blood biochemical indicators in DSS enteritis mouse experiments : (A) ALT and (B) AST.


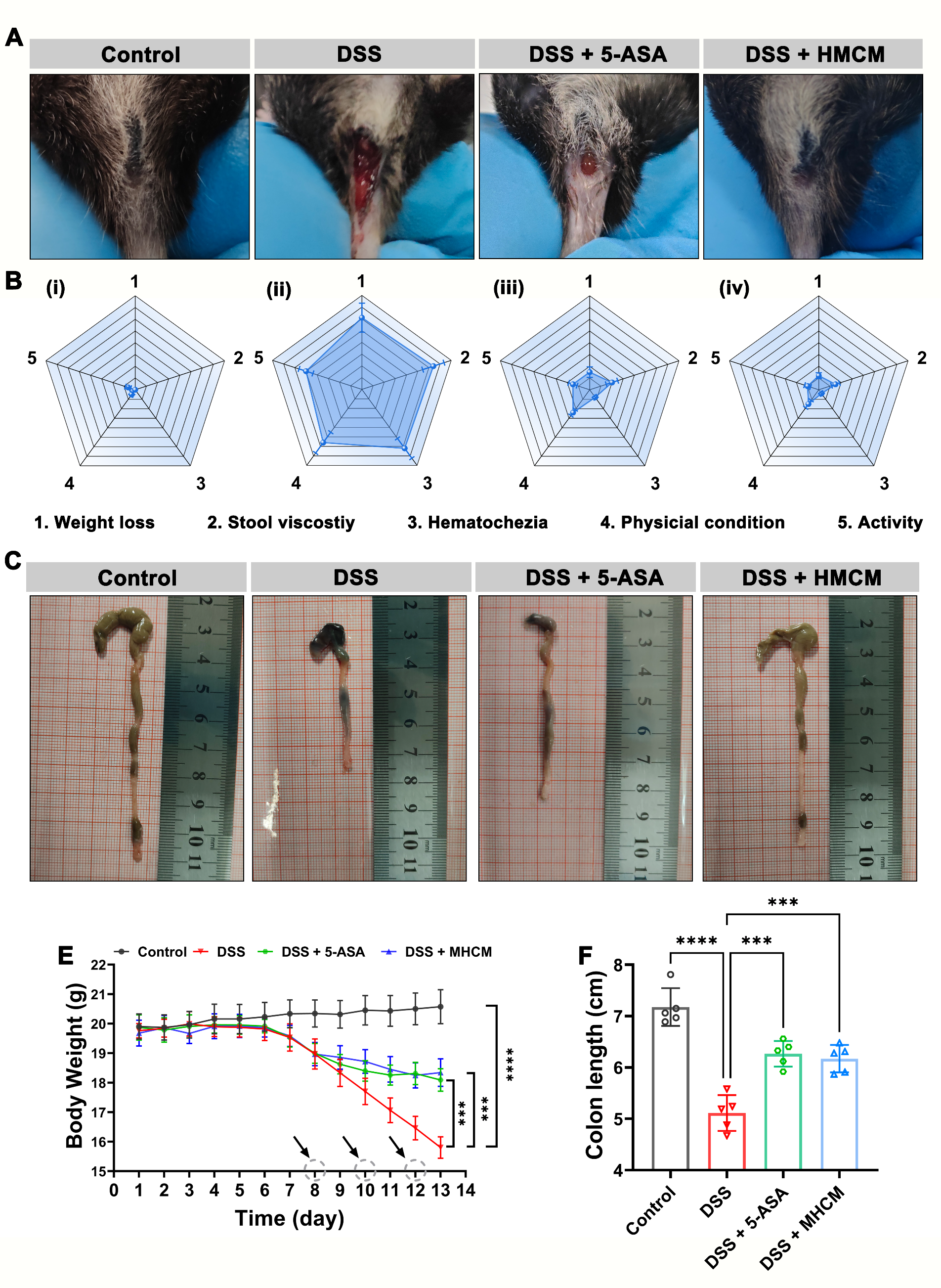


**Fig. S15.** (A) Photographs of the anus of mice in different treatment groups on day 13. (B) IBD disease activity index scores in different treatment groups: i) Control group; ii) DSS group; iii) DSS + 5-ASA group; iv) DSS + HMCM group. (C) Comparison of colon length after treatment of IBD. (E) Daily weight changes in different treatment groups. (F) Quantification of colon length.


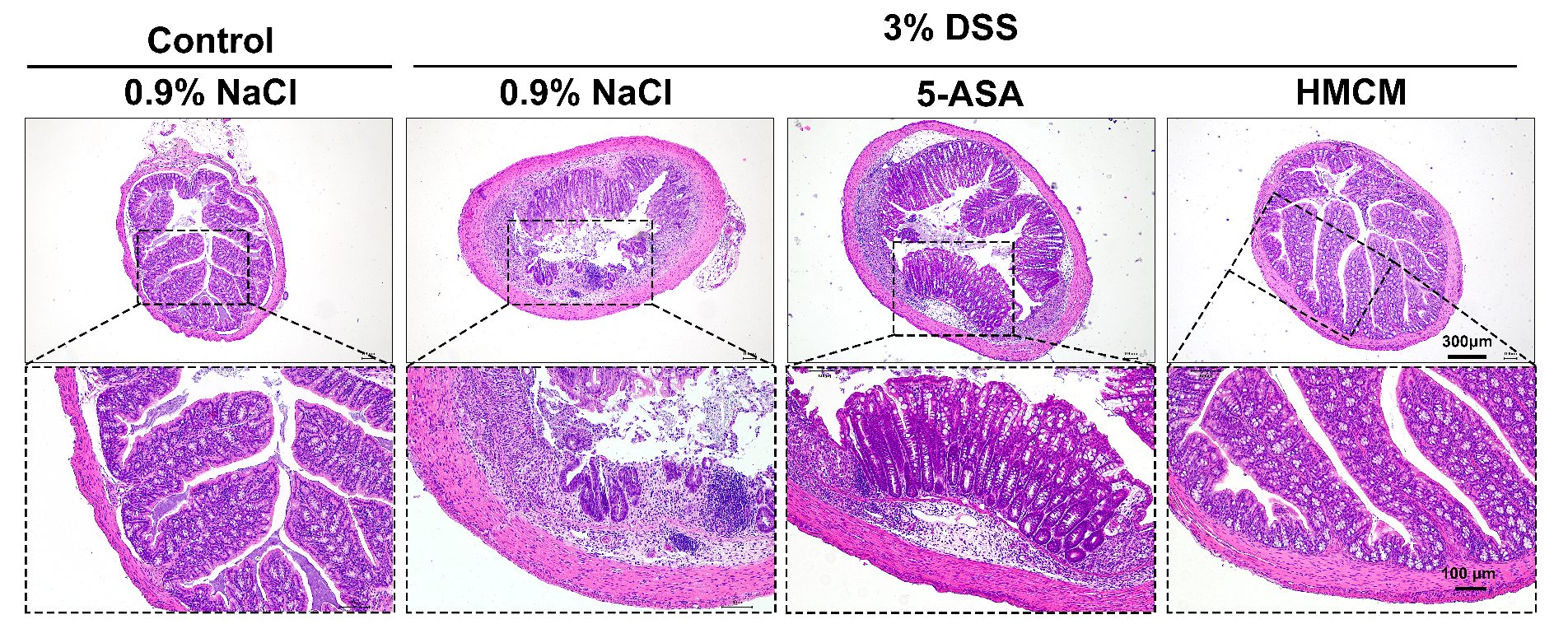


**Fig. S16.** H&E staining


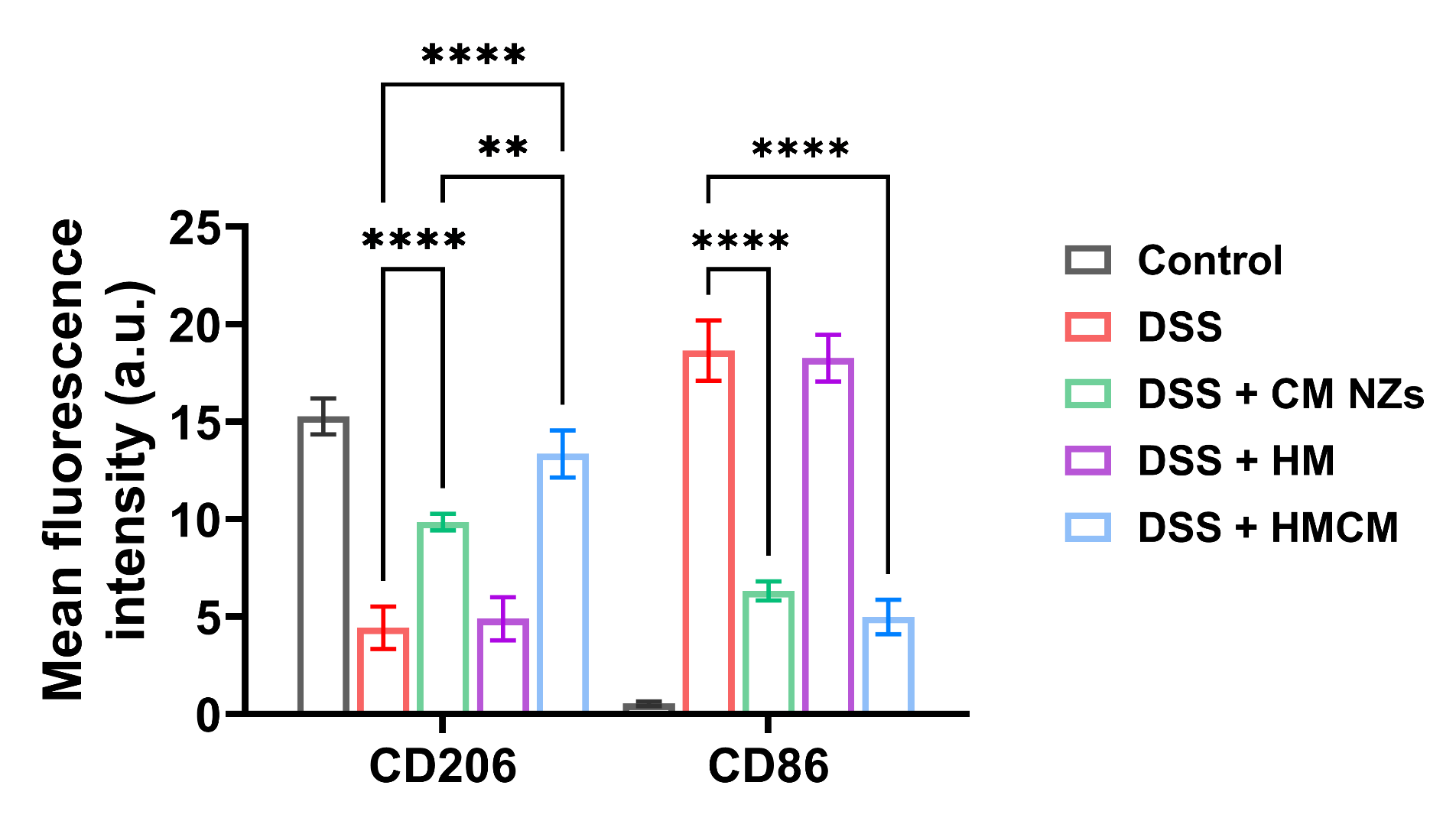


**Fig. S17.** Fluorescence quantitative analysis of CD206 and CD86

**Table S1.** **Scoring system for the status of DSS-induced IBD mice**

| **Score** | **Weight loss**  **(%)** | **Stool**  **viscosity** | **Hematochezia** | **Physicial**  **condition** | **Activity** |
| --- | --- | --- | --- | --- | --- |
| 0 | <5 | normal | nromal | full of vigour | active |
| 1 | 5-15 | loose | mild | lethargy | quiet |
| 2 | 15-25 | sticky | moderate | restlessness | restless |
| 3 | 25-35 | mushy | serious | languish | curled |
| 4 | 35 | liquid | extreme | agonal stage | stiff |

**Table S2.** **The primer sequences for quantitative real-time PCR analysis**

| **Gene Symbol** | **Forward（5’-3’）** | **Reverse（5’-3’）** |
| --- | --- | --- |
| *mHPRT* | GACCGGTCCCGTCATGC | TCATAACCTGGTTCATCATCGC |
| *mTNF-α* | CAGGCGGTGCCTATGTCTC | CGATCACCCCGAAGTTCAGTAG |
| *mIL-1β* | TTCAGGCAGGCAGTATCACTC | GAAGGTCCACGGGAAAGACAC |
| *mIL-6* | CTGCAAGAGACTTCCATCCAG | AGTGGTATAGACAGGTCTGTTGG |
| *mIL-10* | GCCGGGAAGACAATAACTGC | GCCTGGGGCATCACTTCTAC |
| *mGPX4* | CCTCCCCAGTACTGCAACAG | GGCTGAGAATTCGTGCATGG |
| *mCD98* | GGTGAAGGGCCTGGTGTTAG | CTTCCTGGGAGCCCAAAGTG |
| *mSLC7A11* | ATAGCACGAGTGTCAGCTGG | TAGCTGTATAACTCCAGGGACT |
